# Enhancing the Storage Longevity of Apples: The Potential of *Bacillus subtilis* and *Streptomyces endus* as Preventative Bioagents against Post-Harvest Gray Mold Disease, Caused by *Botrytis cinerea*

**DOI:** 10.3390/plants13131844

**Published:** 2024-07-04

**Authors:** Aya Abdelhalim, Yasser S. A. Mazrou, Nabila Shahin, Gabr A. El-Kot, Abdelnaser A. Elzaawely, Hanafey F. Maswada, Abeer H. Makhlouf, Yasser Nehela

**Affiliations:** 1 Department of Agricultural Botany, Faculty of Agriculture, Tanta University, Tanta 31527, Egypt; 2 Business Administration Department, Community College, King Khalid University, Abha 62521, Saudi Arabia; 3 Department of Agricultural Botany, Faculty of Agriculture, Kafr Elsheikh University, Kafr El-Sheikh 33516, Egypt; 4 Department of Agricultural Botany, Faculty of Agriculture, Minufiya University, Shibin El-Kom 6131567, Egypt

**Keywords:** gray mold, *Botrytis*, apple, *Bacillus*, *Streptomyces*, bacterial filtrate, antioxidant, biological control

## Abstract

Gray mold, caused by *Botrytis cinerea* Pers. Fr., is one of the most vital plant diseases, causing extensive pre- and post-harvest losses in apple fruits. In the current study, we isolated and identified two potential endophytic bioagents, *Bacillus subtilis* and *Streptomyces endus*. Both bioagents exhibited a potent fungistatic effect against *B. cinerea* under both in vitro and in planta conditions. Moreover, two experiments were carried out; (i) the first experiment was conducted at room temperature after artificial inoculation with *B. cinerea* to monitor the progression of the infection and the corresponding biochemical responses of the apples. Our in vivo findings showed that the treated *B. cinerea*-infected apple fruits with the cell-free bacterial filtrate of *B. subtilis* and *S. endus* (dipping or wrapping) significantly reduced the rotten area of the treated apple at room temperature. Additionally, *B. subtilis* and *S. endus* enhanced the enzymatic (POX and PPO) and non-enzymatic (phenolics and flavonoids) antioxidant defense machinery in treated apples. (ii) The second experiment focused on the preventive effects of both bioagents over a 90-day storage period at 1 °C of healthy apples (no artificial inoculation). The application of both bacterial filtrates prolonged the storage period, reduced the relative weight loss, and maintained high-quality parameters including titratable acidity, firmness, and total soluble solids of apple fruits under cold storage at 1 °C. The Kaplan–Meier analysis of rotten apples over 90 days during cold storage showed that the treated apples lasted longer than the non-treated apples. Moreover, the lifespan of apple fruits dipped in the culture filtrate of *B. subtilis*, or a fungicide, was increased, with no significant differences, compared with the non-treated apples. The current results showed the possibility of using both bioagents as a safe and eco-friendly alternative to chemical fungicides to control gray mold disease in apples.

## 1. Introduction

Gray mold caused by the ascomycete *Botrytis cinerea* Pers. Fr. (teleomorph, *Botryotinia fuckeliana* (de Bary) Wetzel) infects numerous economically important plant species [1]. *B. cinerea* exhibits a necrotrophic mode of infection, characterized by the maceration of host tissue. It produces asexual macroconidia on the surface of colonized host tissue and forms melanized sclerotia, ensuring long-term survival and facilitating sexual reproduction [2]. *B. cinerea* is a predominant fungal pathogen in many fruits and vegetables including apples, and it causes noxious losses under both the field and storage conditions [3]. The symptoms include various spots on stems, leaves, flowers, and fruits causing stem rot, leaf blight, blossom blight, and fruit rot [4].

Control of *B. cinerea* gray mold relies mainly on the application of chemical fungicides; about 8% of the worldwide fungicide market is dedicated to the management of this pathogen [5]. However, the extensive use of synthetic fungicides in agriculture can lead to resistance in fungal populations and has also generated a series of negative impacts concerning health and environmental hazards [6,7]. Therefore, there is an increasing interest in exploring safer and eco-friendly alternative strategies to the use of chemical fungicides [8]. Various methods used to control post-harvest diseases of fruits and vegetables have previously been documented including cold or controlled atmosphere storage [9], plant extracts, essential oils [10], heating [11], ozone [12], salts [13], biological control [14] and resistance inducers [15].

Biocontrol agents or their derived natural compounds, when applied shortly before or soon after harvest, were found to be relatively successful in controlling post-harvest pathogens [16]. Among these, *Bacillus subtilis* and other related species showed the ability to limit the growth of various phytopathogenic fungi, and they have been highlighted in different studies on account of their safety, wide distribution in various habitats, exceptional survival capability under adverse conditions, and ability to produce growth-promoting substances and a large number of bioactive chemicals [17]. *B. subtilis* repressed the growth and development of gray mold on tomato fruits [18], pomegranate fruits [19], and maize plants [20].

Additionally, there is a raised interest in the utilization of endophytic actinobacteria for agriculture because of their capability to produce a broad spectrum of antibiotics and other secondary metabolites with diverse biological activities [21]. For instance, *Streptomyces endus* OsiSh-2 was isolated from rice and performed excellent biocontrol activity toward *Magnaporthe oryzae* by various mechanisms including antibiotic compounds and siderophore production [22]. In addition, *Streptomyces* sp. sdu1201 and its metabolite, actinomycin D, had great potential as biocontrol agents in the control of strawberry gray mold [23]. However, the effect of both bioagents (*B. subtilis* and *S. endus*) on apple gray mold caused by *B. cinerea* is poorly studied.

Apple gray mold is a disease with two entry routes, i.e., either in the blossom period resulting in blossom end rot or later on through wounds. While we believe that preventive applications are ideal, demonstrating the biocontrol agents’ ability to reduce disease severity post-infection provides a comprehensive understanding of their full potential. This dual approach can offer more flexible and practical solutions for growers dealing with varying infection stages. Our study aimed to evaluate the potential of *B. subtilis* and *S. endus* not only as preventive treatments to inhibit the onset of gray mold caused by *B. cinerea* in apple fruits but also to evaluate how the application of these bioagents can prolong the storage period of apples, maintaining quality parameters such as firmness, titratable acidity, and total soluble solids during cold storage at 1 °C.

In the current study, we isolated and identified two potential bioagents, including *B. subtilis* and *S. endus* for controlling gray mold, caused by *B. cinerea*, in apple fruits. The antifungal activity of both bioagents against *B. cinerea* was investigated in vitro and in vivo using two different application methods (dipping or wrapping) and comparing their efficacy to enhance storage longevity with chemical fungicides. Moreover, to better understand the physio-biochemical mechanism(s) of both bioagents on apple fruits, their effect on the enzymatic and non-enzymatic antioxidant defense machinery in treated apples was determined. Additionally, the impact of both bioagents on extending the storage period and maintaining fruit quality parameters of stored apple fruits during 90 days of cold storage at 1 °C was also examined. We believe that our findings from the current study will establish a foundation for subsequent research focused on the preventative capabilities of *B. subtilis* and *S. endus* in commercial apple storage and management practices. Finally, the ultimate goal of this study is to provide eco-friendly alternative control strategies to reduce the reliance on chemical fungicides and enhance food security and human health.

## 2. Results

### 2.1. Isolation and Identification of Bioagents

**Figure 1:**
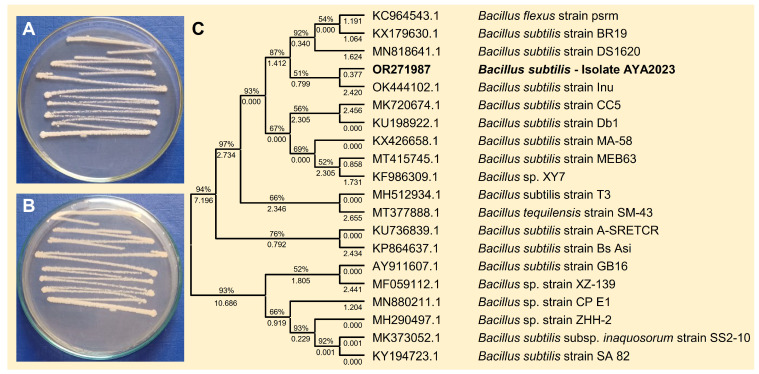
Morphological characterization and molecular identification of the bacterial bioagent *B. subtilis* isolated from healthy apple fruits collected from the local market. (**A**,**B**) The growth and morphological characteristics of the biocontrol agent *B. subtilis* on the nutrient agar medium from the top and the bottom of the Petri dish, respectively, after 2 days of incubation at 37 °C. (**C**) The phylogenetic analysis with the Maximum Likelihood method and Tamura–Nei model based on the sequence similarity of 16S rRNA sequence of *B. subtilis* isolate AYA2023 (GenBank Accession No. OR271987; highlighted in bold) in comparison with 19 reference strains/isolates retrieved from the recently available data from NCBI GenBank. The tree with the highest log likelihood (−28,015.32) is shown. The percentage of trees in which the associated taxa clustered together is shown next to the branches. Initial tree(s) for the heuristic search were obtained automatically by applying Neighbor-Join and BioNJ algorithms to a matrix of pairwise distances estimated using the Tamura–Nei model and then selecting the topology with a superior log likelihood value. The proportion of sites where at least 1 unambiguous base is present in at least 1 sequence for each descendent clade is shown next to each internal node in the tree.

To isolate effective bioagents against *B. cinerea*, a single bacterial isolate was obtained from healthy apple fruits collected from a local market and characterized according to the sequence of the 16S ribosomal RNA gene, in addition to their cultural, morphological, and macroscopic characteristics like *Bacillus* spp. The bacterial isolate was fast-growing, Gram-positive with rod-shaped cells, smooth, convex colonies, and whitish color on the nutrient agar medium after 24 h post incubation at 37 °C (Figure 1A,B), which is consistent with *B. subtilis*. Based on the sequence of the 16S ribosomal RNA gene, the phylogenetic analysis showed that the query sequence showed a high similarity with *B. subtilis* strain Inu (GenBank Accession OK444102.1; Figure 1C) with accepted alignment statistics (query cover = 100%, E value = 0.0, and identity = 94.14%). The new sequence (767 bp) was deposited in the NCBI database and named “*B. subtilis*—AYA2023” (GenBank Accession No. OR271987). Furthermore, the endophytic actinomycetes *S. endus* was previously isolated from soil rhizosphere samples of healthy potato plants as described in our previous study. 

### 2.2. B. subtilis and S. endus Inhibited the Mycelial Growth of B. cinerea

**Figure 2:**
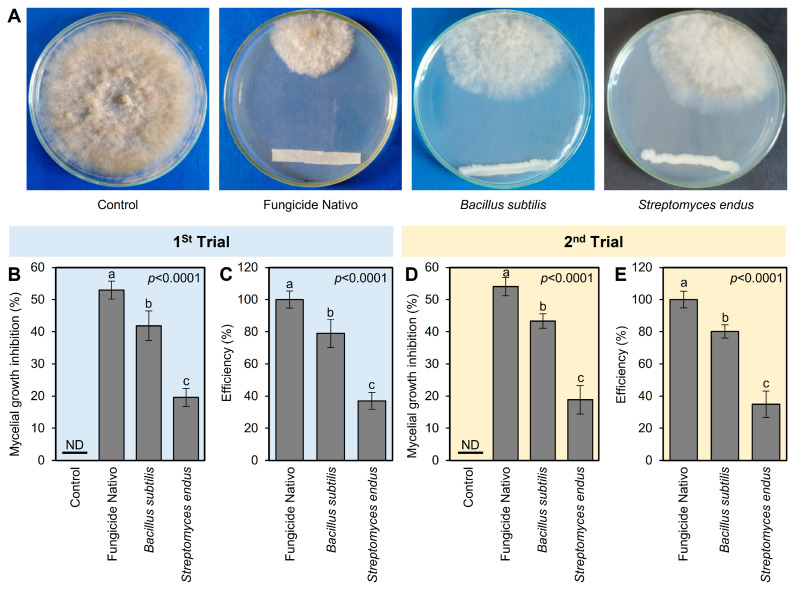
In vitro antifungal activity of the bacterial bioagents *B. subtilis* and *S. endus* against *B. cinerea*, the causal agent of gray mold disease of apple. (**A**) Antifungal activity of *B. subtilis* and *S. endus* against *B. cinerea* using the double culture assay. (**B**,**D**) The mycelial growth inhibition (%) of *B. cinerea* after treatment with *B. subtilis* and *S. endus* using the double culture assay. (**C**,**E**) The efficiency (%) of *B. subtilis* and *S. endus* against *B. cinerea* compared with the commercial fungicide Nativo. Bars denote the means ± standard deviations (means ± SD). Different letters signify statistically significant differences among treatments using Tukey’s Honestly Significant Difference (HSD) Test (*p* < 0.05). ND: Not detected.

The antifungal activities of both bioagents (*B. subtilis* and *S. endus*) against *B. cinerea* were tested in vitro using dual culture assay. In the dual culture plates, both bioagents showed strong fungistatic activity against *B. cinerea* (Figure 2A). In the first trial, the commercial fungicide Nativo showed the highest mycelial growth inhibition (52.96 ± 2.80%); however, *B. subtilis* became the second highest mycelium growth inhibitor (41.85 ± 4.63%) followed by *S. endus* (19.63 ± 2.80%) (Figure 2B). It is worth mentioning that both bioagents (*B. subtilis* and *S. endus*) showed acceptable efficiency (79 and 37%, respectively) compared with the commercial fungicide Nativo (Figure 2C). The second trial confirmed almost the same results in terms of mycelial growth inhibition (%) and efficiency (%) (Figure 2D,E). 

### 2.3. Biological Control of Apple Gray Mold at Room Temperature

#### 2.3.1. *B. subtilis* and *S. endus* Reduced the Apple Gray Mold Disease 

**Figure 3:**
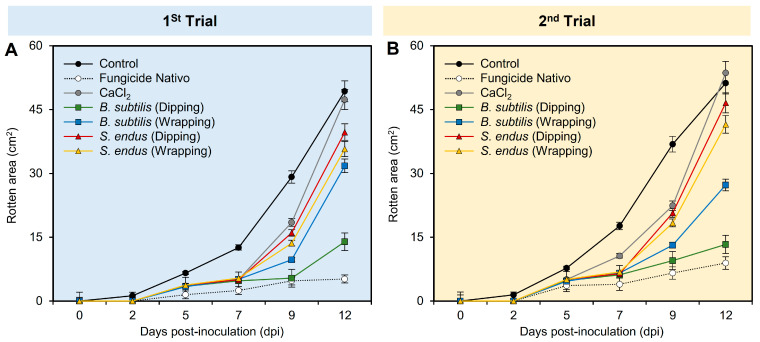
The development of gray mold disease, caused by *B. cinerea* on apple fruits after the treatment with the cell-free culture filtrates of bacterial bioagents *B. subtilis* and *S. endus* at room temperature. (**A**,**B**) Rotten area (cm^2^) of infected Anna apples during 1st trial and 2nd trial, respectively. Dots denote the means, whereas the whiskers donate the standard deviations (means ± SD). Different letters signify statistically significant differences among treatments using Tukey’s Honestly Significant Difference (HSD) Test (*p* < 0.05).

Regardless of the sampling time, while the application of fungicide Nativo significantly reduced the rotten area (02.32 ± 0.32), the apples treated with *B. subtilis* (dipping and wrapping) exhibited the second lowest rotten area (04.60 ± 0.34 and 08.34 ± 0.57, respectively) compared to the non-treated control (16.49 ± 1.11) (Table S1). Similarly, regardless of the treatments, the treated apples had the lowest rotten area at 0 and 2 dpi (0.0 ± 0.0 and 0.21 ± 0.02, respectively) compared to 12 dpi (31.87 ± 2.16). In general, the rotten area of treated apples increased over time during two separate experiments (Figure 3A,B and Table S1). However, apple fruits treated with fungicide Nativo or culture filtrates of *B. subtilis* significantly showed smaller rotten areas compared with other treatments, particularly at 12 dpi. 

#### 2.3.2. *B. subtilis* and *S. endus* Stimulated the Enzymatic Antioxidant Defense Machinery of Apple Fruits

**Figure 4:**
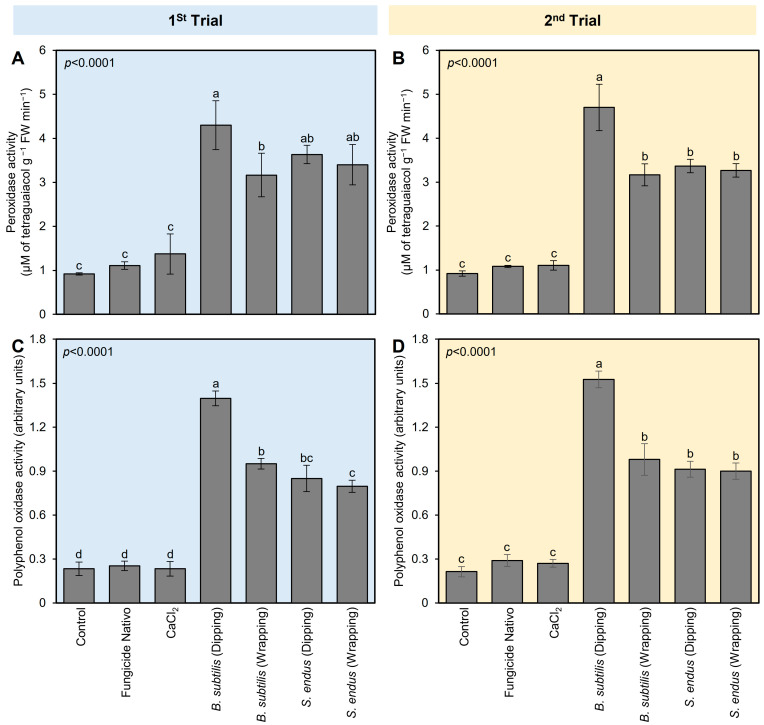
The effect of cell-free culture filtrates of *B. subtilis* and *S. endus* on the enzymatic antioxidant defense system of *B. cinerea*-infected apple fruits at room temperature. (**A**,**C**) The peroxidase activity (μM of tetra guaiacol g^−1^ FW min^−1^) during 1st trial and 2nd trial, respectively. (**B**,**D**) Polyphenol oxidase activity (arbitrary units) during 1st trial and 2nd trial, respectively. Bars denote the means ± standard deviations (means ± SD). Different letters signify statistically significant differences among treatments using Tukey’s Honestly Significant Difference (HSD) Test (*p* < 0.05).

To better understand the physio-biochemical mechanism(s) of both bioagents on apple fruits, the effect of *B. subtilis* and *S. endus* on the enzymatic and non-enzymatic antioxidant defense machinery (as expressed by the enzymatic activity of POX and PPO) in treated apples was examined (Figure 4). Generally, the application of cell-free filtrates of *B. subtilis* and *S. endus* considerably enhanced the enzymatic activity of POX and PPO in treated apples at 72 hpi (Figure 4). Dipping apples in culture filtrates of *B. subtilis* before storage increased the activity of POX (4.30 ± 0.55 × 10^−2^ μM of tetra guaiacol g^−1^ FW min^−1^; Figure 4A) and PPO (1.39 ± 0.05 arbitrary units; Figure 4B) compared with non-treated control (0.92 ± 0.02 × 10^−2^ μM of tetra guaiacol g^−1^ FW min^−1^, 0.23 ± 0.04 arbitrary units, respectively). The second trial confirmed almost the same findings (Figure 4C,D).

#### 2.3.3. *B. subtilis* Enhanced the Non-Enzymatic Antioxidant Defense Machinery of Apple Fruits

**Figure 5:**
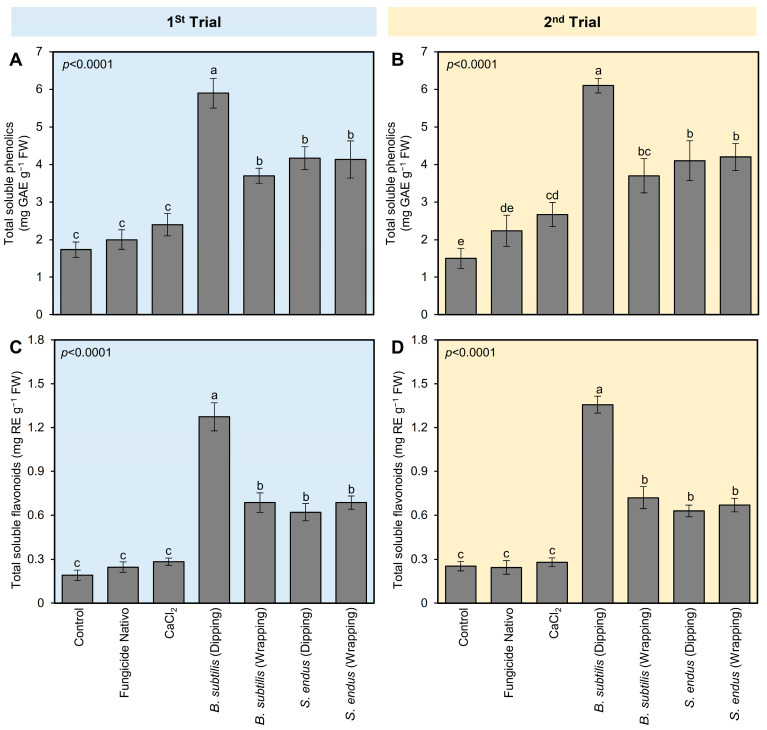
The effect of cell-free culture filtrates of *B. subtilis* and *S. endus* on the non-enzymatic antioxidant defense system of *B. cinerea*-infected apple fruits at room temperature. (**A**,**C**) Total soluble phenolics (mg GAE g^−1^ FW) during 1st trial and 2nd trial, respectively. (**B**,**D**) Total soluble flavonoids (mg RE g^−1^ FW) during 1st trial and 2nd trial, respectively. Bars denote the means ± standard deviations (means ± SD). Different letters signify statistically significant differences among treatments using Tukey’s Honestly Significant Difference (HSD) Test (*p* < 0.05).

Similar to the profile of POX and PPO, all tested treatments enhanced the profile of total soluble phenolics and flavonoids of *B. cinerea*-infected apples at 72 hpi (Figure 5). Briefly, dipping apples in culture filtrates of *B. subtilis* before storage significantly augmented the total soluble phenolics (5.90 ± 0.40 mg GAE g^−1^ FW; Figure 5A) and flavonoids (1.27 ± 0.09 mg RE g^−1^ FW; Figure 5B) compared with non-treated control at 72 hpi. *B. subtilis* [dipping] was followed by *B. subtilis* [wrapping], *S. endus* [dipping], and *S. endus* [wrapping], which were higher than controls with no significant differences between them. The second trial confirmed almost the same results (Figure 5C,D).

### 2.4. Biological Control of Apple Gray Mold under Cold Storage (1 °C)

Building upon the above-mentioned experiments conducted at room temperature for only 12 days, the current study extends the investigation to cold storage conditions for 90 days. To simulate real-world post-harvest scenarios, apples were subjected to prolonged cold storage (at 1 °C) to assess the long-term efficacy of *B. subtilis* and *S. endus* and to determine the optimal application methods (wrapping vs. dipping) in the long run. Generally, both bioagents significantly enhanced the storage characteristics such as storage period and lifespan, and maintained the fruit quality parameters including relative weight loss, acidity, firmness, and total soluble solids (TSSs) under these extended cold storage conditions. By integrating these results with those obtained at room temperature, they provide comprehensive insights into the effectiveness of biological control agents in controlling gray mold and preserving apple quality over an extended storage period.

#### 2.4.1. *B. subtilis* and *S. endus* Diminish the Gray Mold Disease and Enhance the Storage Characteristics of Apples under Cold Storage (1 °C) Conditions

**Figure 6:**
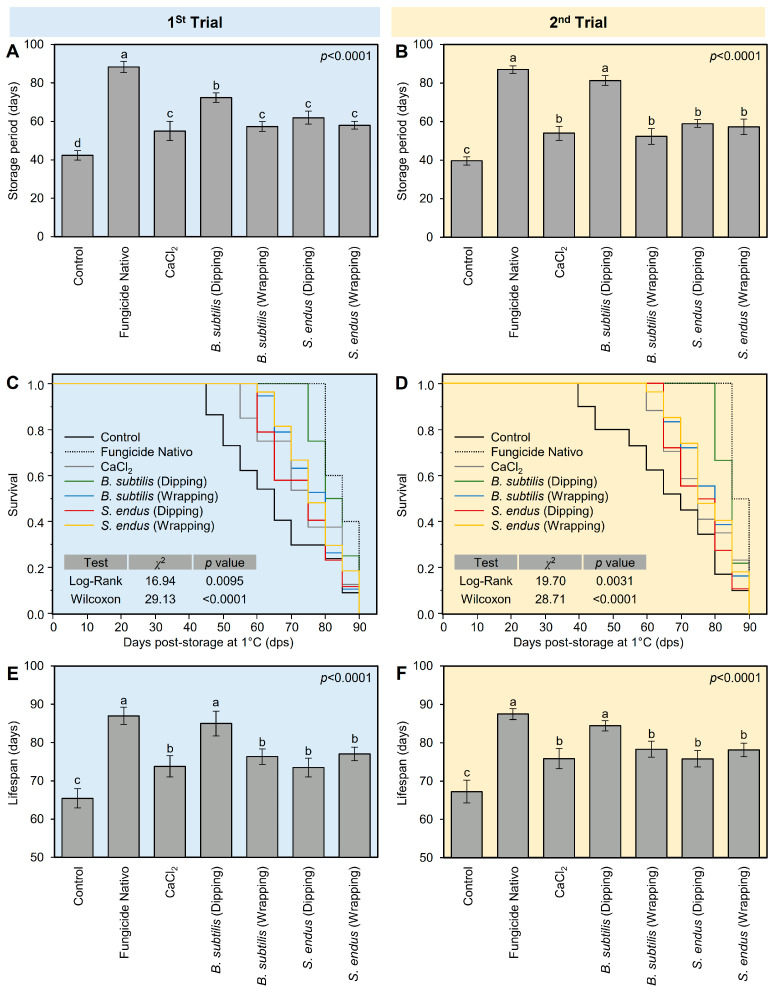
The effect of cell-free culture filtrates of *B. subtilis* and *S. endus* on storage characteristics of apple fruits under cold storage. (**A**,**B**) Effect of cell-free culture filtrates of *B. subtilis* and *S. endus* on storage period (days) of apple fruits during 1st trial and 2nd trial, respectively. (**C**,**D**) Kaplan–Meier analysis of rotten apples over 90 days after cell-free culture filtrates of *B. subtilis* and *S. endus* during cold storage during 1st trial and 2nd trial, respectively (*n* = 30). *p* values and χ^2^ of Log-Rank and Wilcoxon tests (presented in the upper right corner of the graph) were used for statistical comparisons among the curves. (**E**,**F**) Lifespans of apple fruits treated with cell-free culture filtrates of *B. subtilis* and *S. endus* over 90 days under cold storage (*n* = 30) during 1st trial and 2nd trial, respectively. Bars denote the means ± standard deviations (means ± SD). Different letters signify statistically significant differences among treatments using Tukey’s Honestly Significant Difference (HSD) Test (*p* < 0.05).

In general, all treatments prolonged the storage period of treated apples compared with the non-treated control. The fungicide-treated apples had the longest storage period (88.33 ± 2.89 days), followed by apples dipped in the cell-free filtrate of *B. subtilis* (72.33 ± 2.52 days), both of which were significantly higher than the non-treated control (42.33 ± 2.52 days) (Figure 6A). It is worth mentioning that other treatments including *B. subtilis* (wrapping), *S. endus* (dipping), and *S. endus* (wrapping) were almost similar to the commercial control (CaCl_2_), with no significant differences among them. The same trend was noticed in the second trial; however, dipped in the cell-free filtrate of *B. subtilis* was comparable to fungicide-treated apples, with no significant differences between them (Figure 6B). 

Moreover, Kaplan–Meier analysis (Figure 6C,D) was conducted to better understand the relationship between apple gray mold and storage time under cold conditions (1 °C) and to determine the expected duration of time for apples to rot. It is worth mentioning that, although the first diseased apples were noticed at 40 days post-storage (dps) in the control treatment, the first infected apples in *B. subtilis* (dipping) treatment were observed at 80 dps (Figure 6C). In the second trial, almost the same trend was noticed (Figure 6D). The Kaplan–Meier analysis of rotten apples over 90 days during cold storage showed that the event history of bio-treated apples was significantly higher than the non-treated apples (controls) in the 1st trial (n = 30; χ^2^ = 16.94 and 29.13 for Log-Rank and Wilcoxon tests, respectively; *p* = 0.0095 and *p* < 0.0001 for both tests; Figure 6C) and 2nd trial (n = 30; χ^2^ = 19.70 and 28.71 for Log-Rank and Wilcoxon tests, respectively; *p* = 0.0031 and *p* < 0.0001 for both tests; Figure 6D). 

Simlarly, in both trials, the lifespan of fungicide-treated apples (87.00 ± 2.24 days) was similar to those dipped in the bacterial filtrate of *B. subtilis,* with no significant differences between them, but both were higher than other treatments (Figure 6E,F). However, *B. subtilis* (wrapping), *S. endus* (dipping), and *S. endus* (wrapping) were almost similar to the commercial control (CaCl_2_), with no significant differences among them, and all were higher than the non-treated control.

#### 2.4.2. *B. subtilis* and *S. endus* Maintain the Quality Indices and Physiological Properties of Apples under Cold Storage (1 °C) Conditions

- Relative weight loss (%)

**Figure 7:**
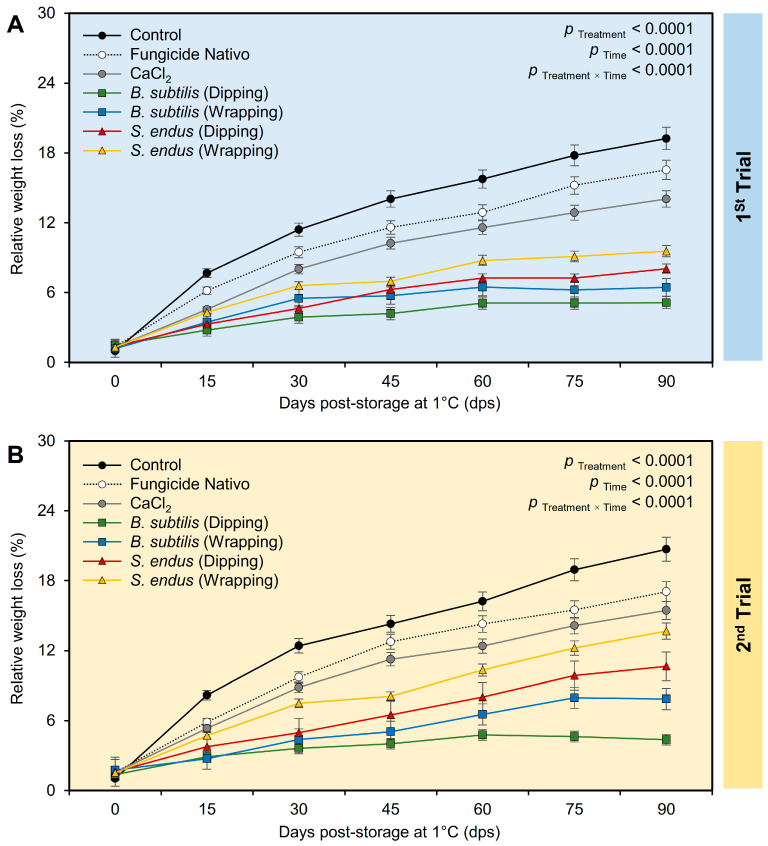
The effect of cell-free culture filtrates of *B. subtilis* and *S. endus* on relative weight loss of apple fruits over 90 days storage under cold storage. (**A**,**B**) Relative weight loss (%) during 1st trial and 2nd trial, respectively. Bars denote the means ± standard deviations (means ± SD).

Regardless of the time points, applying the bacterial filtrate of *B. subtilis* and *S. endus* significantly reduced the relative weight loss of treated apples (Table S2). It is worth mentioning that the apples dipped in the culture filtrate of *B. subtilis* had the lowest weight loss (3.96 ± 0.32% and 3.66 ± 0.26%, in the 1st and 2nd trials, respectively) compared with the untreated control (12.42 ± 0.22% and 13.11 ± 0.24%, in the 1st and 2nd trials, respectively). The treated apples had the lowest weight loss at 15 dps (4.61 ± 0.56% and 4.78 ± 0.67%, in the 1st and 2nd trials, respectively) compared with those stored for the total 90 days (11.29 ± 0.21% and 12.82 ± 0.23%, in the 1st and 2nd trials, respectively) (Table S2). Although, the percentage of relative weight loss of treated apples was increased over the time of the experiment (Figure 7A), dipping apples before storage in the culture filtrates of *B. subtilis* significantly reduced the relative weight loss over the time course. Both negative (non-treated) and positive controls (fungicide and CaCl_2_) showed a higher reduction in the weight of storage apples than other treatments. In the 2nd trial, almost the same trend was noticed (Figure 7B).

b. Titratable acidity

**Figure 8:**
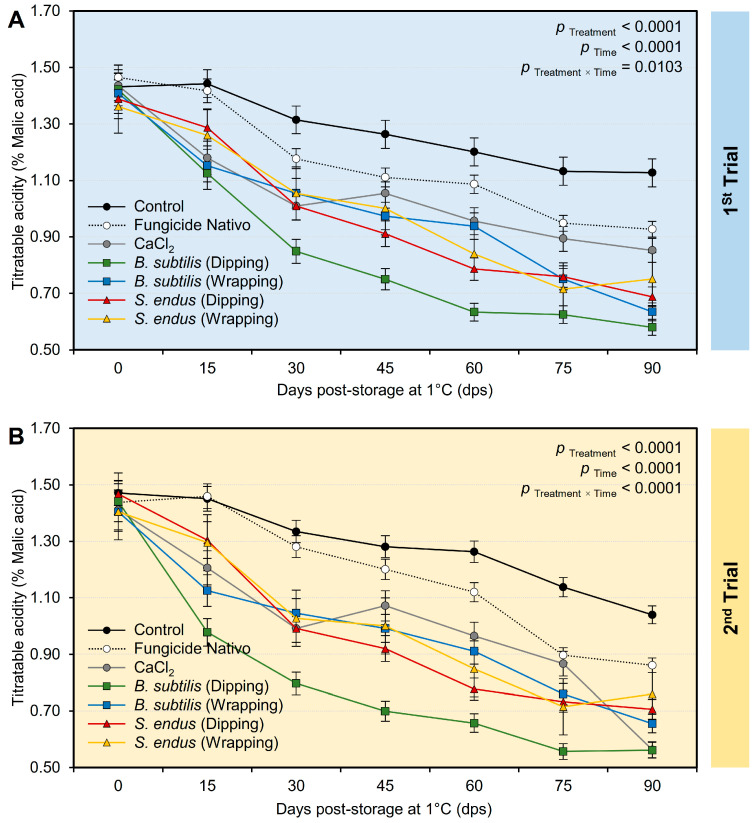
The effect of cell-free culture filtrates of *B. subtilis* and *S. endus* on titratable acidity of apple fruits over 90 days storage under cold storage. (**A**) Titratable acidity (%) during 1st trial and (**B**) titratable acidity (%) during 2nd trial. Bars denote the means ± standard deviations (means ± SD).

Generally, applying both *B. subtilis* and *S. endus* culture filtrates significantly decreased the acidity of treated apples (Table S3) in both trials, regardless of the time points. It is worth mentioning that apples dipped in a cell-free filtrate of *B. subtilis* had the lowest acidity (0.85 ± 0.09% and 0.81 ± 0.08%, in the 1st and 2nd trials, respectively) compared with the untreated control (1.27 ± 0.06% and 1.28 ± 0.03%, in the 1st and 2nd trials, respectively). It is worth mentioning that the treated apples had the lowest acidity at 90 days (0.79 ± 0.08% and 0.74 ± 0.08%, in the 1st and 2nd trials, respectively) compared to the initial time (1.42 ± 0.05% and 1.43 ± 0.07%, in the 1st and 2nd trials, respectively) regardless of the treatments. Although the acidity of treated apples showed downward over the time of the experiment (Figure 8A,B), both negative (non-treated) and positive controls (fungicide and CaCl_2_) showed lower reduction than other treatments. Additionally, dipping apples before storage in the culture filtrates of *B. subtilis* resulted in a significant reduction in acidity over the experiment course with the lowest level at 90 days. Almost the same acidity profile was observed during both trials (Figure 8A,B).

c. Firmness

**Figure 9:**
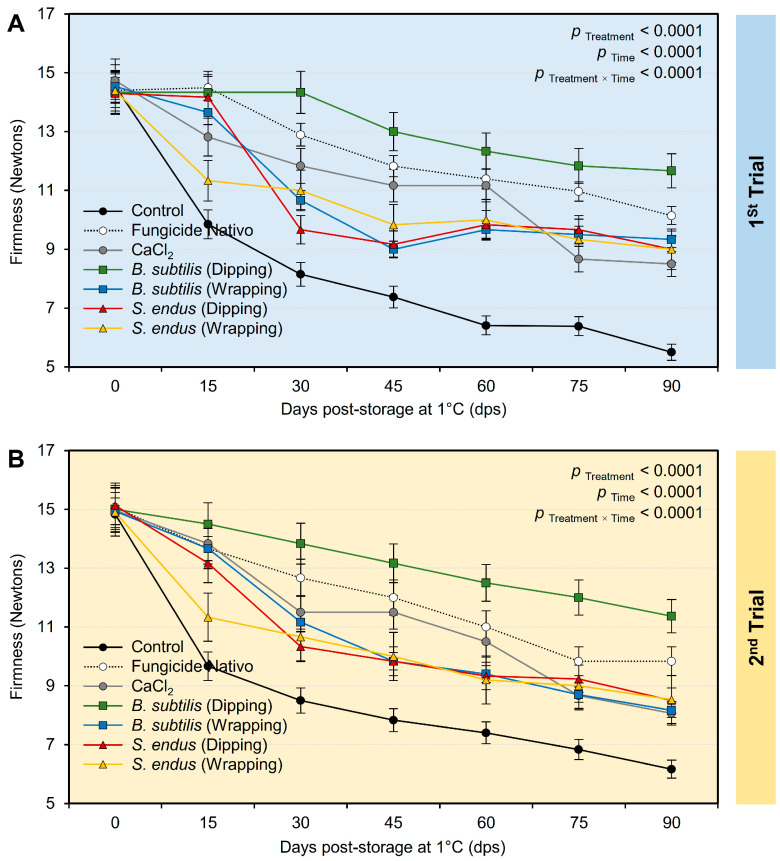
The effect of cell-free culture filtrates of *B. subtilis* and *S. endus* on firmness of apple fruits over 90 days storage under cold storage. (**A**) Firmness (Newton) during 1st trial and (**B**) firmness (Newton) during 2nd trial. Bars denote the means ± standard deviations (means ± SD).

Regardless of the time points, the treatment with the cell-free bacterial filtrate *B. subtilis* and *S. endus* significantly maintained the firmness of treated apples (Table S4). Interestingly, apples dipped in a cell-free filtrate of *B. subtilis* had the highest firmness (13.12 ± 0.70 and 13.20 ± 0.48 Newtons, in the 1st and 2nd trials, respectively) compared with the untreated control (8.31 ± 0.55 and 8.75 ± 0.39 Newtons, in the 1st and 2nd trials, respectively). Moreover, the firmness of treated apples (notwithstanding the treatment) was dropped from 14.46 ± 0.30 and 14.98 ± 0.47 Newtons, in the 1st and 2nd trials, respectively, at the initial time to 9.02 ± 0.97 and 8.66 ± 0.51 Newtons, in the 1st and 2nd trials, respectively, after 90 days of storage under cold conditions (1 °C) (Table S4). Although the firmness of stored apples significantly decreased over the time of the experiment (Figure 9A,B), dipping apples in the culture filtrates of *B. subtilis* before storage significantly maintained the firmness of treated apples over the experiment course. It is worth mentioning that both positive controls (Nativo fungicide and CaCl_2_) had a compromised effect compared with the non-treated controls and showed lower reduction than other treatments.

d. Total soluble solids (TSSs)

**Figure 10:**
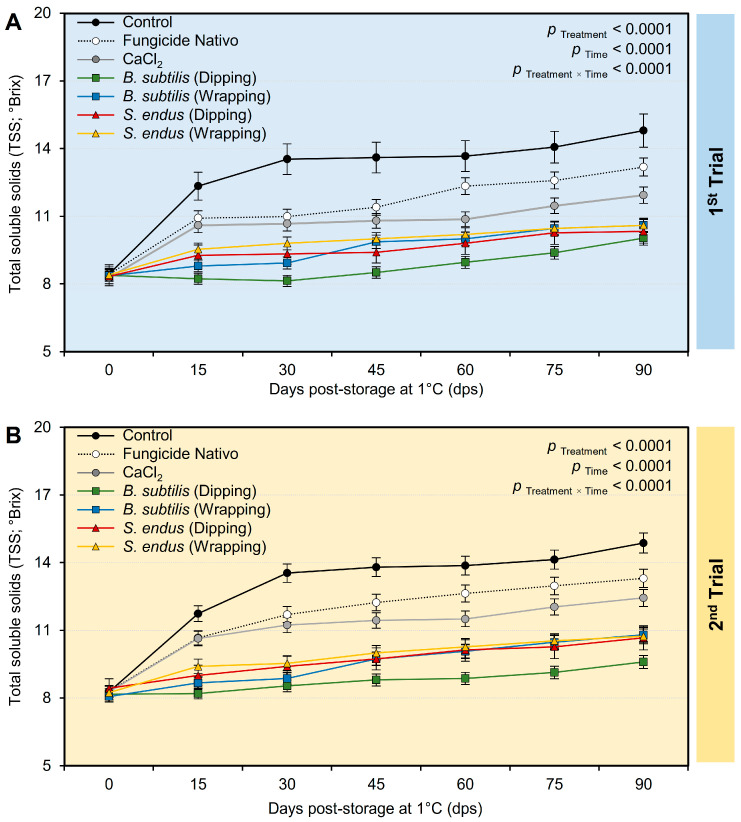
The effect of cell-free culture filtrates of *B. subtilis* and *S. endus* on the total soluble solids of apple fruits over 90 days storage under cold storage. (**A**) Total soluble solids (°Brix) during 1st trial and (**B**) total soluble solids (°Brix) during 2nd trial. Bars denote the means ± standard deviations (means ± SD).

Generally, and regardless of the treatments, the total soluble solids (TSSs) significantly increased from 8.37 ± 0.23 and 8.25 ± 0.19 °Brix, in the 1st and 2nd trials, respectively, at the initial time to 11.64 ± 0.37 and 11.77 ± 0.22 °Brix, in the 1st and 2nd trials, respectively, at 90 dps under cold conditions (1 °C) (Table S5). However, applying the cell-free culture filtrate of *B. subtilis* and *S. endus* via dipping or wrapping significantly maintained the TSS of treated apples. It is worth mentioning that apples dipped in the cell-free filtrate of *B. subtilis* had the lowest TSSs (08.80 ± 0.55 and 08.76 ± 0.28 °Brix, in the 1st and 2nd trials, respectively) compared with the non-treated control (12.92 ± 0.40 and 12.89 ± 0.28 °Brix, in the 1st and 2nd trials, respectively) (Table S5). Although the TSSs in the water-treated control dramatically increased at 15 days post storage and till the end of the experiment, the TSSs of apples dipped in the bacterial filtrate of *B. subtilis* maintained almost the same levels with a slight increase at 60, 75, and 90 dps (Figure 10A). Similarly, the data from the second trial confirmed the same profile (Table S5 and Figure 10B).

## 3. Discussion

The post-harvest journey of apples faces numerous challenges worldwide, particularly being susceptible to various phytopathogenic microbes [24,25,26]. Several phytopathogenic fungi were previously reported as major pathogens in apple storage facilities, leading to significant economic losses due to decay and reduced quality. These phytopathogenic fungi include, but are not limited to, *B. cinerea*, *Rhizopus stolonifer*, *Penicillium* spp., *Alternaria* spp., *Aspergillus* sp., *Mucor* sp., *Stemphylium* spp., *Colletotrichum* spp., *Lasiodiplodia* sp., *Fusarium* spp., and *Trichothecium roseum* [24,26]. However, gray mold, caused by the fungus *B. cinerea*, is one of the most significant post-harvest diseases if not effectively managed [25,26].

Accordingly, managing post-harvest diseases, in general, and gray mold, in particular, is a critical issue that challenges apple production worldwide. Traditionally, chemical control has been employed as the most effective strategy for gray mold control for years. However, its efficacy diminishes since the frequent and large-scale use of numerous fungicides leads to increasingly serious “3R” problems (Resistance, Resurgence, and Residue) [27]. Searching for efficient eco-friendly management alternatives is necessary. Considering these challenges, the exploration of sustainable, effective, and eco-friendly control alternatives is required. Biological control presents a promising avenue for managing gray mold while maintaining apple quality. Several bioagents showed promising potential in managing different post-harvest diseases, including bacteria [28,29,30,31,32,33], yeasts [34,35,36], and fungi [37]. For instance, several *Bacillus* species, including, but not limited to, *B. amyloliquefaciens* RS-25, *B. licheniformis* MG-4, *B. subtilis* Z-14, *B. subtilis* Pnf-4, *B. halotolerans*, and *B. mojavensis* D50, showed a strong antifungal activity and efficiently inhibited *B. cinerea* on post-harvest tomato, strawberry, and grapefruit [30,31,32].

Briefly, the cell-free culture filtrate of both *B. subtilis* and *S. endus* significantly inhibited the growth of *B. cinerea* and reduced the development of gray mold disease in vitro. The antagonistic activity of *B. subtilis* against various phytopathogens such as *B. cinerea*, *Fusarium oxysporum*, *Rhizoctonia solani*, *Sclerotinia sclerotiorum*, *Phytophthora capsici*, *Pythium ultimum*, and *Ralstonia solanacearum* [38] was previously documented [39]. Likewise, *Streptomyces* strains exhibited a strong biocontrol efficacy against *Alternaria alternata* [40], *R. solani* [41], *Phytophthora drechsleri* [42], and *Verticillium dahliae* [43]. In addition, endophytic *S. endus* has potential as a biocontrol agent against *Magnaporthe oryzae* [44]. However, further studies are needed to investigate the role of extracellular metabolites of *B. subtilis* and *S. endus* against *B. cinerea.*

Numerous explanations were suggested to understand the antifungal activity of both bacterial strains. For example, the ability of these bacteria to produce a variety of bioactive molecules with a potential inhibitory action against phytopathogens [45] and siderophore production [46]. Furthermore, some *Bacillus* spp. can produce a variety of enzymes, e.g., β-1,3-glucanase, cellulase, and protease, which play an important role in the cell wall degrading of fungi [47,48]. Furthermore, the mode of action of bioagents such as *Bacillus* sp. may be due to the production of different compounds, such as n- hexadecanoic acid (palmitic acid), 2-heptenal, 2-octenal, and octadecenoic acid (oleic acid), in a higher percentage, which has a direct effect on hyphae and sporangia of *B. cinerea* [19]. Moreover, it was reported recently that several *Bacillus* strains including *B. amyloliquefaciens* RS-25, *B. licheniformis* MG-4, *B. subtilis* Z-14, and *B. subtilis* Pnf-4 which, have inhibitory properties against *B. cinerea,* can produce volatile organic compounds (VOCs) and show potent cellulase and protease activities, but no chitinase activity [30]. It was previously reported that the fungistatic activity of *S. endus* against *Magnaporthe oryzae* is mediated by the presence of active enzymes and secondary metabolites such as cellulase, protease, gelatinase, antibiotics, siderophore, indole-3-acetic acid, and 1-amino-cyclopropane-1-carboxylate deaminase [44]. Additionally, the antagonistic activity of *S. endus* against *F. oxysporum*, *A. solani*, and *Streptomyces scabies* is associated with its ability to produce antibiotics [49]. 

In the current study, it is obvious that both bacterial culture filtrates significantly reduced the disease development of gray mold on treated apple fruits compared with the non-treated fruits. This might be due to the induction of the antioxidant defense system in treated apples. The antioxidant defense system in plants is a complex network designed to protect cells from oxidative damage caused by reactive oxygen species (ROS). This system includes various enzymatic and non-enzymatic components that work together to detoxify ROS and repair oxidative damage [50]. The main antioxidant enzymes include superoxide dismutase (SOD), catalase (CAT), ascorbate peroxidase (APX), glutathione peroxidase (GPX), glutathione reductase (GR), and peroxidase (POX) [50,51]. It is worth mentioning that POX detoxifies hydrogen peroxide to maintain redox homeostasis [51]. Moreover, polyphenol oxidase (PPO) is an enzyme that plays a significant role in the plant’s defense mechanisms, particularly in response to wounding and pathogen attacks. It is involved in the oxidation of phenolic compounds to quinones [51], which subsequently polymerize to form brown pigments. Although PPO is not a primary component of the antioxidant defense system that directly detoxifies reactive oxygen species (ROS), it contributes to the overall defense strategy of plants in several ways. 

Remarkably, our results demonstrated that the enzymatic activities of both POX and PPO in treated apples were significantly enhanced due to the application of culture filtrates of *B. subtilis* and *S. endus*. Moreover, phenolics and flavonoids are considered non-enzymatic antioxidants, which play an important role in plant disease resistance [52]. In the present study, the levels of total soluble phenolics and flavonoids were increased in treated apple fruits. These results confirm the crucial role of *B. subtilis* and *S. endus* in supporting the antioxidant defense machinery in *B. cinerea*-infected apple fruits to mitigate the damage caused by the reactive oxygen species (ROS) and preserve their homeostasis. 

Increasing or enhancing the activity of oxidizing enzymes such as PPO, POX, and other related enzymes in apple fruits using bioagent bacterial filtrates can have both desirable and undesirable effects, depending on the context and the specific goals of post-harvest management and storage. Desirable effects include (i) enhancing defense against pathogens since the higher activity of oxidizing enzymes can improve the fruit’s natural defense mechanisms against fungal pathogens. The formation of antimicrobial quinones and the strengthening of cell walls can reduce the incidence of post-harvest diseases [28,29,31,32,53,54]. (ii) Extending the shelf life due to enhanced enzyme activity can lead to a slower rate of microbial growth, potentially extending the shelf life of the apples under certain conditions [28,29,32,55,56]. On the other hand, undesirable effects include (i) increasing browning on treated apples where the enhanced activity of oxidizing enzymes, particularly PPO, can lead to increased enzymatic browning of apple tissues. This browning occurs when phenolic compounds are oxidized to quinones, which then polymerize to form brown pigments. This can significantly reduce the aesthetic and market quality of fresh apples and negatively affect consumer acceptance and satisfaction [28,31,32,50,53,54]. (ii) Oxidation reactions can sometimes produce off-flavors or alter the natural flavor profile of apples, making them less palatable, and sometimes can lead to the breakdown of cell walls and other structural components, potentially affecting the texture of the fruit [28,29,32,52,55,56]. Therefore, careful selection and application of bioagents, along with complementary post-harvest strategies, are necessary to optimize the benefits and minimize the drawbacks [53,54,55,56]. Anyway, further studies are required to better understand the long-term effect of the application of culture filtrates of *B. subtilis* and *S. endus* on enzymatic and non-enzymatic antioxidants and their effect on fruit quality.

The increase in phenolic content is a common plant response to biotic stress, which involves the upregulation of the phenylpropanoid pathway. Phenolics play crucial roles in reinforcing the cell wall and acting as antimicrobial agents [50,51]. These enzymes use phenolics as substrates to produce quinones and other compounds that participate in defense reactions, further enhancing the structural integrity of the cell walls [51]. It is worth mentioning that the treatment of apple fruits with cell-free bacterial filtrates of *B. subtilis* or *S. endus,* leading to higher activities of PPO and POX, along with increased levels of phenolics and flavonoids, can affect the fruit’s firmness and durability in several ways. Briefly, the increased activity of PPO and POX can lead to the formation of lignin-like polymers in the cell walls, which strengthens the cell wall structure [57,58,59]. Moreover, the elevated levels of phenolics and flavonoids contribute to the cross-linking of cell wall polysaccharides, enhancing the rigidity and firmness of the fruit [58,59]. Likewise, the antimicrobial properties of phenolics and flavonoids can reduce the activity of pectinases and cellulases produced by pathogens, which are responsible for cell wall breakdown [58,60] and could induce the defense-related enzymes and compounds that help in maintaining cell wall integrity by inhibiting pathogen-induced cell wall degradation [57,58,59,60]. Collectively, our findings suggest that treating apples with cell-free bacterial filtrates of *B. subtilis* or *S. endus* enhances PPO and POX activity and increases phenolics and flavonoid levels, which in turn might lead to improved firmness and durability. The enhanced firmness results from strengthened cell wall integrity and reduced degradation by pathogens. These changes improve the resistance of the apples to decay, maintain their textural quality, and delay senescence, thereby extending their shelf life and improving their marketability.

Likewise, total soluble solids (TSSs) are an important quality parameter for fruits, including apples. TSSs are often used as an indicator of sweetness and overall flavor, impacting consumer preference and marketability [61]. The effect of maintained TSS levels in stored apples, particularly concerning higher activities of PPO and POX and elevated levels of phenolics and flavonoids, can help preserve the flavor quality of the apples [62,63]. Consistent TSS levels indicate stable fruit quality during storage, suggesting that the apples are less susceptible to over-ripening and spoilage [62,63,64]. While PPO and POX mainly contribute to the oxidative browning process and defense mechanisms, their activity does not directly impact TSS levels. However, their role in maintaining cell wall integrity and reducing pathogen load indirectly supports the retention of TSSs by preserving fruit quality and delaying senescence [57,58,59,60,62,63]. Furthermore, elevated levels of phenolics and flavonoids enhance the antioxidant capacity of the fruit, protecting cellular components from oxidative damage. This protection helps maintain metabolic processes that are crucial for preserving TSS levels.

In the current study, the results showed that apple fruits treated with the bacterial culture filtrates of *B. subtilis* and *S. endus* extended the storage period of treated apples compared with the non-treated control. In addition, the results showed that the life of treated apples with bacterial culture filtrates of *B. subtilis* and *S. endus* was significantly longer than the non-treated apples by the Kaplan–Meier analysis of rotten apples over 90 days during cold storage. It was previously indicated that the incidence of rot was significantly lower in pomegranates treated with bacterial culture filtrates when compared with the control (non-treated fruits) and reported the possibility of using culture filtrates of *B. subtilis* in plant disease management including the control of post-harvest diseases of fruits caused by *Penicillium* sp., *Aspergillus* sp., *Fusarium* sp., and other fungal pathogens and to prolong storage period of fruits such as apples [65]. 

Our findings indicate that the application of *B. subtilis* and *S. endus* reduced the relative weight loss of treated apples. These results are in agreement with [19] who reported that the treatment with bacterial bioagents had significantly decreased weight loss of pomegranate fruits. Weight loss percentage increased with the prolonging of the storage period of “Anna” apple fruits during cold storage at 0 °C [66]. In addition, the weight loss of wax apple and guava treated with an ε-PL fermentation broth of *Streptomyces* sp. increased continuously with storage [67]. Loss of weight in fresh fruits and vegetables is mainly due to water loss as a result of evaporation and transpiration, plus the amount of dry matter lost by fruit respiration [68]. 

It was reported previously that titratable acidity decreased during the storage period and was explained by the CO_2_ effect of inhibiting the activity of decarboxylating enzymes in the respiratory cycle, thus contributing to maintaining acidity [69]. Additionally, malic acid, the main organic acid found in apple fruit, is a main substrate for aerobic respiration, which usually decreases during cold storage. Ref. [70] found that fruit treated with *Bacillus siamensis* strain maintained very high levels of total soluble solids and titratable acidity compared with the control fruit. 

It is worth mentioning that these findings are in agreement with our current study where we showed that the application of *B. subtilis* and *S. endus* decreased the acidity and maintained the total soluble solids of treated apples under cold storage. Additionally, our study revealed that treatment with *B. subtilis* and *S. endus* maintained the firmness of treated apples. In agreement with these findings, apple fruit firmness was significantly increased by *Bacillus* and *Microbacterium* applications compared with the control [71]. Notably, treatment with bioagents has been reported to stimulate the biosynthesis of secondary metabolites, such as phenylpropanoids, within the host plant [72]. Moreover, biocontrol agents are an effective strategy in inducing disease resistance against phytopathogens. Previous studies indicated that *Bacillus* sp., for instance, can boost resistance against phytopathogens by regulating the transcription of genes or enzymes associated with plant defense mechanisms [73].

## 4. Materials and Methods

### 4.1. Plant Materials

The commercial cultivar “Anna” of apple (*Malus domestica* Borkh L.) was used as experimental plant material throughout this study. Briefly, apple fruits were harvested from a commercial orchard located in Kafr Al-Mansoura, Shubra Al-Namla, Tanta (30.7865° N, 31.0004° E), Gharbia governorate, at the commercial ripening stage. Fruits were collected from similar trees. The selected trees were 25 years old, grown in sandy clay soil under a flood irrigation system, and planted 5 × 5 m apart. The apple orchard was watered every 12 days, and all other cultural practices were performed according to the recommendations of the Ministry of Agriculture, Egypt. Approximately 350 healthy apples were collected. The collected apple fruits were almost similar in terms of size (horizontal diameter = 60 ± 5 mm, and vertical height = 65 ± 5 mm), shape, gloss, color, and free from any disease, external damage, mechanical injury, defects, scratches, wounds, and/or bruises. Immediately after harvesting, healthy apple fruits were directly transported to the laboratory of the Department of Agricultural Botany, Faculty of Agriculture, Tanta University, air-cooled for 24 h in a cooling chamber, and then used for further experiments. 

### 4.2. The Phytopathogenic Fungus B. cinerea 

The aggressive strain of *B. cinerea* (GenBank Accession No. OR730908) was used throughout this study. The used isolate was previously isolated from a rotten apple, morphologically characterized, and molecularly identified as *B. cinerea* [26] based on the sequence of large subunit ribosomal RNA of the ITS region. To prepare the spore suspension of *B. cinerea*, a 5 mm fungal plug was transferred to a PDA medium and incubated at 25 ± 2 °C for 7–10 days. Subsequently, spores were rubbed from the agar surface with a sterile glass rod. The high-density spore suspension was passed through two layers of chess cloth, and then, the number of spores per mL was counted using a hemacytometer and diluted with sterile water to a spore inoculum concentration of 1 × 10^5^ spore.mL^−1^.

### 4.3. Bacterial Bioagents

The endophytic actinomycetes *S. endus* was isolated from soil rhizosphere samples of healthy potato plants as described in our previous study and identified based on its cultural, morphological, and macroscopic characteristics as described in our previous study [74]. On the other hand, *B. subtilis* was isolated in the current study from healthy apple fruits. *B. subtilis* was initially identified based on its cultural, morphological, and macroscopic characteristics. Then, the identification was molecularly confirmed based on the 16S rRNA sequencing. The assembled query sequence was subsequently compared with different bacterial genera (Table S6) that are available in GenBank of the national center for biotechnology information website (NCBI, http://www.ncbi.nlm.nih.gov/gene/; accessed on 28 October 2023) using the Nucleotide–Nucleotide Basic Local Alignment Search Tool (BLASTn) [75,76]. 

Subsequently, two cell-free filtrates from *B. subtilis* and *S. endus* were prepared to evaluate their antifungal activity against *B. cinerea*. Initially, a small amount of the bacterial culture was transferred into a new nutrient-rich broth medium using a flame-sterilized cooled loop and then incubated at 25 ± 2 °C for 2–3 days on a shaker at 150 rpm until the late logarithmic or early stationary phase. Once the bacterial cultures reached the desired growth phase, they were centrifuged at 7000 rpm for 10 min to separate the bacterial cells from the culture medium. To ensure that the bacterial filtrate is cell-free, the liquid medium supernatant was further filtered through a membrane-based syringe filter (pore size = 0.45 μm, diameter = 33 mm; Sartorius Stedim Biotech GmbH, Göttingen, Germany) to eliminate any remaining viable bacterial cells or contaminants and stored at −4 °C for further experiments.

### 4.4. In Vitro Antifungal Activity of B. subtilis and S. endus

The in vitro antifungal activity of *B. subtilis* (AYA2023) and *S. endus* was evaluated using the dual culture technique. Both bacterial strains were streaked individually, each at 1 cm from the outer edge of Petri dishes filled with PDA medium, and then placed in an incubator set at 28 ± 2 °C for 48 h. Then, a 6 mm fungal plug of *B. cinerea* obtained from a freshly growing culture was put on the opposite side of the plates. Inoculated plates streaked with a line of sterile deionized water were used as a negative control. As a positive control, a 0.4 × 3.5 cm bar of filter paper was saturated with 10 µL of the fungicide Nativo (Tebuconazole 50% + Trifloxystrobin 25%) at the recommended dose (0.25 g.L^−1^) that acts as a protective and curative fungicide, and it was placed at the edge. Subsequently, all plates were incubated at 25 ± 2 °C for 7 days or until the mycelial growth of *B. cinerea* entirely covered the control plates. The inhibition zone was determined by measuring the distance between the bioagent bacteria and the outer edge of the fungal mycelium as described previously [77]. Mycelial growth inhibition was calculated using Equation (1):

(1) $$Mycelial\;growth\;inhibition\;(\%)=\frac{C-T}{C}\times 100$$

Here, C signifies the mycelial growth in the control, and T indicates the mycelial growth in the treatments.

### 4.5. In Vivo Antifungal Activity of B. subtilis and S. endus

#### 4.5.1. Biological Control of Gray Mold Disease at Room Temperature

Generally, seven treatments (Table 1) were tested in the current study on Anna apples during storage at room temperature. Apple fruits were surface sterilized by double immersion in (10%) sodium hypochlorite solution for 3 min and then washed with sterilized distilled water and allowed to dry at room temperature under sterile conditions. All fruits were wounded (with holes of 5 mm diameter and 4 mm depth) on one location of the fruit skin and inoculated with 20 μL of 1 × 10^5^ spore.mL^−1^ of *B. cinerea* suspension previously prepared as described in Section 4.2. Infected apples were divided into seven groups and subjected to the following treatment:

The commercial fungicide Nativo (Tebuconazole 50% + Trifloxystrobin 25%; acts as a protective and curative fungicide) and CaCl_2_ were used as a positive control, while sterilized water was used as a negative control. Each treatment contains three biological replicates, with 5 fruits per replicate packed in carton boxes (40 cm length × 30 cm width × 10 cm height). Apple fruits were stored at room temperature (25 ± 2 °C) for 12 days post-inoculation (dpi). Fruit decay was visually evaluated during storage when the rot zone outside the wounded area on the fruit was visible, and then, the rot diameter of each fruit was measured at 2, 5, 7, 9, and 12 dpi. The whole experiment was repeated twice using the same experimental design. 

#### Antioxidant Enzymes

The enzymatic activities of two antioxidant enzymes including polyphenol oxidase (PPO) and peroxidases (POX) were colorimetrically determined using a UV-160 spectrophotometer (Model SM1200 UV-Vis, AZZOTA Corporation, Claymont, DE, USA). Briefly, 0.5 g of fruit samples were collected at 72 h post-inoculation (hpi) and then homogenized using a pre-frozen mortar and pestle, along with 3 mL of 50 mM Tris buffer (pH 7.8) containing 1 mM EDTA-Na2 and 7.5% polyvinylpyrrolidone (PVP). Following homogenization, the mixture was centrifuged for 20 min at 11,269× *g* under cooling (4 °C). The extract of fresh apple fruits was used as a crude enzyme extract for the following analysis. For POX activity assessment, the reaction mixture consisted of 2.2 mL of 100 mM sodium phosphate buffer (pH 6.0), 100 μL of guaiacol, 100 μL of 12 mM H_2_O_2_, and 10 μL of crude enzyme extract, and then, the increase in the absorption at 436 nm (A_436_) was measured [78]. However, PPO activity was determined according to the method of Malik and Singh [79]. Briefly, the reaction mixture contained 3 mL catechol solution (0.01 M), newly prepared in 0.1 M phosphate buffer (pH 6.0), and then, 100 μL of enzyme extract was added to activate the reaction. Changes in the absorbance at 495 nm (A_495_) were recorded every 30 s for 3 min. 

#### Total Soluble Phenolic Compounds

The total soluble phenolics (TSPs) were assessed using the Folin–Ciocalteu reagent [80] with slight modifications. Briefly, treated apple fruits were collected for phenolic extraction at 72 hpi. Approximately, 100 mg of fruit tissue was homogenized and mixed with 20 mL of methanol 80% for 24 h. For TSP assessment, 200 μL of methanolic extract was mixed with 1000 μL of Folin–Ciocalteu reagent (10%) and vortexed for 30 s, then 800 μL of 7.5% sodium carbonates (*w*/*v*) was added, shaken well, and then incubated for 30 min at room temperature to allow color development. After incubation, the absorbance of the resulting blue-colored solution was measured at 765 nm using a spectrophotometer (Model SM1200 UV-Vis, AZZOTA Corporation, Claymont, DE, USA). A standard curve of known concentrations of gallic acid was used for the quantification of TSP, which was expressed as mg gallic acid equivalents per gram fresh weight (mg GAE g^−1^ FW).

#### Total Soluble Flavonoids

The quantification of total soluble flavonoids (TSFs) followed the methodology outlined by Djeridane et al. (2006) [81] with slight modifications. Briefly, 200 μL of methanolic extract derived from apple fruit was mixed with an equal volume of aluminum chloride solution (2% in methanol), vigorously mixed, and then incubated at room temperature for 15 min. Absorbance was measured at 430 nm using the same spectrophotometer described above. TSFs were subsequently reported as milligrams of rutin equivalents per gram of fresh weight (mg RE g^−1^ FW).

#### 4.5.2. Biological Control of Gray Mold Disease during Cold Storage at 1 °C 

Healthy and unwounded Anna apple fruits were chosen and washed by dipping in tap water for 10 min to remove any specks of dirt, then treated by dipping for 4 min in 3% (*w*/*v*) calcium chloride (CaCl_2)_ solution, and then air dried. Subsequently, the fruits were randomly divided into seven groups, and then, each group was treated individually with one of the treatments as mentioned in Table 1, but under cold storage at 1 °C. During cold storage, each treatment was maintained in carton boxes containing 2 g of potassium permanganate (KMnO_4_) as an ethylene absorber and then stored at 1 °C and 90% RH for 90 days post storage (dps).

#### Disease Assessment

a. Storage period

The storage period of each treatment was determined according to the number of days in which fruits stayed intact during the storage period at 1 °C and 90% RH, and the maximum storage period reached 90 days.

b. Disease incidence (DI)

The number of infected fruits was counted every five days post-inoculation (dpi) to determine the disease incidence (DI) percentage using Equation (2):

(2) $$Disease\;incidence\;\left(DI;\%\right)=\frac{Number\;of\;infected\;fruits}{Total\;number\;of\;fruits}\times 100$$

To better understand the relationship between rot symptoms and storage time, DI was expressed using Kaplan–Meier analysis (event history analysis). The fruit survivorship was recorded every five days until the end of the experiment (90 days). Fruits found with a necrotic lesion (>0.5 cm) were counted as infected (decayed). Survival analysis was carried out using the Kaplan–Meier method [82] and their associated lifespan (days) was obtained. 

#### The Quality Indices and Physiological Properties

- Weight loss (%)

Weight loss was approximated as a percentage of the initial weight at 0, 15, 30, 45, 60, 75, and 90 days post-inoculation (dpi). For this purpose, the weight of the fruits was determined at time zero (before storage), during (every 15 days), and at the end of storage at 1 °C. The percentage of weight loss was calculated using Equation (3):

(3) $$Fruit\;weight\;loss\;\left(\%\right)=\frac{Initial\;weight\;at\;a\;specific\;time}{Initial\;weight}\times 100$$

b. Total soluble solids (TSSs)

The total soluble solids (TSSs) were determined in the juice of treated apples at 0, 15, 30, 45, 60, 75, and 90 dpi. Briefly, fresh apple juice was extracted from each biological sample by pressing through a muslin cloth, and the juice was collected in a clean beaker. TSSs were determined in the juice using a portable refractometer, 0–32 scale (ATAGO N-1E, Minato-ku, Tokyo, Japan), by placing a small volume of apple juice on the prism surface, and then, the readings were expressed in degrees Brix (°Brix) [83]. Samples were measured at room temperature in triplicates, and care was taken to ensure the cleanliness of the equipment and the accuracy of the readings.

c. Titratable acidity (TA)

The titratable acidity (TA) was determined by titrating 5 mL of filtered juice samples, obtained as described above, with sodium hydroxide (NaOH) solution (0.1 N) in the presence of three drops of phenolphthalein as an indicator of the endpoint (until a faint pink color persisted for at least 30 s) and then expressed as a percentage of malic acid [83]. Correction for any background acidity was determined from a blank titration with distilled water.

d. Flesh firmness

Flesh firmness was measured by using a portable fruit firmness tester (FT-327, QA Supplies LLC, Norfolk, VA, USA) equipped with an 11 mm cylindrical stainless steel plunger tip. Briefly, after the calibration of the tester, according to the manufacturer’s instructions, and the skin of the tested area was removed, the probe of the firmness tester was placed onto the selected testing area of the apple. Then, gentle pressure was applied till the probe penetrated the flesh to a predetermined depth. Firmness readings were taken from both sides of the fruit and expressed in Newton (N).

### 4.6. Statistical Analysis

All in vitro experiments were performed in a completely randomized design, whereas the cold storage experiment was laid out in a split-plot design comprising seven treatment combinations in the main plots (control, fungicide Nativo, CaCl_2_, *B. subtilis* (dipping), *B. subtilis* (wrapping), *S. endus* (dipping), and *S. endus* (wrapping)) and seven-time points in subplots (0, 15, 30, 45, 60, 75, and 90 dpi). All experiments were repeated twice with at least three biological replicates for each treatment. All data were statistically analyzed according to the analysis of variance technique (ANOVA), followed by Tukey’s Honestly Significant Difference (HSD) Test as a post hoc analysis based on the *p*-value of treatments (*p* _Treatment_ < 0.05), time (*p* _Time_ < 0.05), and their interaction (*p*_Treatment × Time_ < 0.05). Furthermore, event history analysis (aka survival analysis) was carried out using the Kaplan–Meier method. *p* values and χ^2^ of Log-Rank and Wilcoxon tests were used for statistical comparisons among the curves.

## 5. Conclusions

Generally, our findings highlight the potential importance of *B. subtilis* and *S. endus* and their filtrates against *B. cinerea*, the causal agent of apple gray mold. Our findings showed that both bacteria significantly inhibited the mycelial radial growth of *B. cinerea* and showed fungistatic activities in vitro. Moreover, the cell-free filtrates of *B. subtilis* and *S. endus* diminished the expansion of gray mold. This is probably due to their fungistatic properties and/or due to the stimulation of the antioxidant defense machinery of *B. cinerea*-infected apples. Additionally, culture filtrates of *B. subtilis* and *S. endus* prolonged the storage period and maintained high-quality indices and physiological properties of apples under cold storage (1 °C) conditions. Both application methods (dipping or wrapping) in formulations of the culture filtrates of both bioagents have shown promising results in reducing gray mold incidence during storage at room temperature or under cold storage conditions (1 °C). The results obtained in this study suggest that both bioagents might be eco-friendly alternatives to reduce the utilization of chemical fungicides entirely or partially to maintain human health and food security. By integrating biological control methods into post-harvest management practices, growers can mitigate the impact of gray mold while ensuring high-quality apples for consumers. Moreover, the compatibility of these agents with organic farming practices further enhances their appeal for sustainable disease management.

## Figures and Tables

**Table 1:** Treatments used in this study.

Treatment	The Way of Exposition	Time of Exposition	Solution/Treatment
Control	Dipping	3 min	Sterilized distilled water
Fungicide Nativo	Dipping	3 min	Commercial fungicide Nativo at the recommended dose (0.25 g.L^−1^)
CaCl_2_	Dipping	3 min	3% CaCl_2_ solution
*B. subtilis*	Dipping	3 min	Cell-free filtrate of *B. subtilis.*
	Wrapping	Stored together till the end of the experiment	Tissue papers saturated with a cell-free filtrate of *B. subtilis*
*S. endus*	Dipping	3 min	Cell-free filtrate of *S. endus.*
	Wrapping	Stored together till the end of the experiment	Tissue papers saturated with a cell-free filtrate of *S. endus*

## Data Availability

The original contributions presented in the study are included in the article/Supplementary Material, and further inquiries can be directed to the corresponding authors.

## Supplementary Materials

The following supporting information can be downloaded at: https://www.mdpi.com/article/10.3390/plants13131844/s1, Table S1: In vitro effect of the cell-free culture filtrates of bacterial bioagents *B. subtilis* and *S. endus* on the rotten area (cm^2^) caused by *B. cinerea*, the causal agent of gray mold disease on apple fruits at room temperature; Table S2: Effect of the cell-free culture filtrates of bacterial bioagents *B. subtilis* and *S. endus* on the relative weight loss (%) caused by *B. cinerea*, the causal agent of gray mold disease on apple fruits over 90 days storage under cold storage; Table S3: Effect of the cell-free culture filtrates of bacterial bioagents *B. subtilis* and *S. endus* on the titratable acidity (%) caused by *B. cinerea*, the causal agent of gray mold disease on apple fruits over 90 days storage under cold storage; Table S4: Effect of the cell-free culture filtrates of bacterial bioagents *B. subtilis* and *S. endus* on the firmness (Newton) caused by *B. cinerea*, the causal agent of gray mold disease on apple fruits over 90 days storage under cold storage; Table S5: Effect of the cell-free culture filtrates of bacterial bioagents *B. subtilis* and *S. endus* on the total soluble solids (°Brix) caused by *B. cinerea*, the causal agent of gray mold disease on apple fruits over 90 days storage under cold storage; Table S6: Sequences from different bacterial genera that produce significant alignment with 16S ribosomal RNA gene from *B. subtilis*.
