# Supplementary material for: Enhancing the Storage Longevity of Apples: The Potential of Bacillus subtilis and Streptomyces endus as Preventative Bioagents against Post-Harvest Gray Mold Disease, Caused by Botrytis cinerea
Source: Plants (Basel). 2024 Jul 4;13(13):1844. doi: 10.3390/plants13131844 (PMC11243874; doi:10.3390/plants13131844)
Supplement: Supplementary file 1 [file plants-13-01844-s001.zip › plants-3038764-supplementary.pdf]

**Table S1.** ***In vitro*** **effect of the cell-free culture filtrates of bacterial bioagents** ***B. subtilis*** **and** ***S. endus*** **on the rotten area ($cm^2$) caused by** ***B. cinerea,*** **the causal agent of gray mold disease on apple fruits at room temperature.**

| Treatment | Days post-inoculation (dpi) | | | | | | Mean±SD |
|---|---|---|---|---|---|---|---|
| | 0 | 2 | 5 | 7 | 9 | 12 | |
| **1st Trial** | | | | | | | |
| Control | 0.00±0.00 k | 1.27±0.25 jk | 6.60±0.62 hi | 12.6±0.98 fg | 29.17±2.30 d | 49.30±2.52 a | 16.49±1.11 a |
| Fungicide "Nativo" | 0.00±0.00 k | 0.00±0.00 k | 1.50±1.32 jk | 2.48±0.08 ijk | 04.73±0.25 ij | 05.20±0.26 ij | 02.32±0.32 f |
| $CaCl_2$ | 0.00±0.00 k | 0.00±0.00 k | 3.53±0.20 ijk | 5.08±0.63 ij | 18.49±3.53 e | 47.37±3.35 a | 12.41±1.29 b |
| *B. subtilis* (Dipping) | 0.00±0.00 k | 0.00±0.00 k | 3.48±0.23 ijk | 4.77±0.25ij | 05.40±0.10 ij | 13.97±1.44 f | 04.60±0.34 e |
| *B. subtilis* (Wrapping) | 0.00±0.00 k | 0.00±0.00 k | 3.38±0.03 ijk | 5.15±0.13 ij | 09.70±0.75 gh | 31.80±2.52 cd | 08.34±0.57 d |
| *S. endus* (Dipping) | 0.00±0.00 k | 0.00±0.00 k | 3.78±0.26 ijk | 5.00±0.50 ij | 16.03±1.55 ef | 39.70±1.71 b | 10.75±0.67 c |
| *S. endus* (Wrapping) | 0.00±0.00 k | 0.00±0.00 k | 3.75±0.25 ijk | 5.38±0.38 ij | 13.60±1.11 fg | 35.77±3.30 bc | 09.75±0.84 c |
| Mean±SD | 0.00±0.00 e | 0.18±0.04 e | 3.72±0.42 d | 5.78±0.42 c | 13.87±1.37 b | 31.87±2.16 a | |
| $p_{Treatment}$ | < 0.0001 | | | | | | |
| $p_{Time}$ | < 0.0001 | | | | | | |
| $p_{Treatment×Time}$ | < 0.0001 | | | | | | |
| **2nd Trial** | | | | | | | |
| Control | 0.00±0.00 o | 1.45±0.17 no | 7.77±0.54 jkl | 17.66±1.98 g | 36.88±1.74 d | 51.22±2.37 a | 19.16±1.13 a |
| Fungicide "Nativo" | 0.00±0.00 o | 0.00±0.00 o | 3.63±0.42 mno | 03.96±0.47 mn | 06.60±1.04 klm | 08.95±1.35 jk | 03.86±0.55 g |
| $CaCl_2$ | 0.00±0.00 o | 0.00±0.00 o | 5.05±0.51 lmn | 10.63±0.81 hij | 22.41±1.81 f | 53.65±1.72 a | 15.29±0.81 b |
| *B. subtilis* (Dipping) | 0.00±0.00 o | 0.00±0.00 o | 4.79±0.25 lmn | 06.18±0.27 klm | 09.50±0.60 ijk | 13.30±0.95 h | 05.63±0.35 f |
| *B. subtilis* (Wrapping) | 0.00±0.00 o | 0.00±0.00 o | 4.68±0.03 lmn | 06.60±0.14 klm | 13.12±2.00 hi | 27.30±2.36 e | 08.62±0.76 e |
| *S. endus* (Dipping) | 0.00±0.00 o | 0.00±0.00 o | 5.11±0.28 lm | 06.43±0.54 klm | 20.72±1.44 fg | 46.58±2.91 b | 13.14±0.86 c |
| *S. endus* (Wrapping) | 0.00±0.00 o | 0.00±0.00 o | 5.08±0.27 lmn | 06.85±0.41 klm | 18.36±1.35 g | 41.53±1.86 c | 11.97±0.65 d |
| Mean±SD | 0.00±0.00 e | 0.21±0.02 e | 5.16±0.33 d | 8.33±0.66 c | 18.23±1.43 b | 34.65±1.93 a | |
| $p_{Treatment}$ | < 0.0001 | | | | | | |
| $p_{Time}$ | < 0.0001 | | | | | | |
| $p_{Treatment×Time}$ | < 0.0001 | | | | | | |

Values denote the means ± standard deviations (means ± SD) of three biological replicates.

Different letters signify statistically significant differences among treatments using Tukey's Honestly Significant Difference (HSD) Test ($p < 0.05$).

**Table S2. Effect of the cell-free culture filtrates of bacterial bioagents *B. subtilis* and *S. endus* on the relative weight loss (%) caused by *B. cinerea*, the causal agent of gray mold disease on apple fruits over 90 days storage under cold storage.**

| Treatment | Days post- storage (dps) | | | | | | | Treatments (Mean±SD) |
|---|---|---|---|---|---|---|---|---|
| | 0 | 15 | 30 | 45 | 60 | 75 | 90 | |
| **1st Trial** | | | | | | | | |
| Control | 0.00±0.00z | 7.69±0.41 klm | 11.41±0.13 g | 14.05±0.12 e | 15.76±0.4 cd | 17.79±0.29 b | 19.24±0.13 a | 12.42±0.22 a |
| Fungicide "Nativo" | 0.00±0.00z | 6.17±0.27 n-i | 9.47±0.12 hi | 11.6±0.3 g | 12.9±0.18 f | 15.22±0.39 d | 16.54±0.29 c | 10.46±0.24 b |
| $CaCL_2$ | 0.00±0.00z | 4.55±0.66 t-w | 8.03±0.45 jkl | 10.25±0.17 h | 11.59±0.22 g | 12.86±0.23 f | 14.04±0.25 e | 8.95±0.3 c |
| *B. subtilis* (Dipping) | 0.00±0.00z | 2.78±0.93 y | 3.89±0.24 v-y | 4.2±0.44 u-x | 5.1±0.2 r-u | 5.1±0.1 q-u | 5.14±0.14 p-u | 3.96±0.32 g |
| *B. subtilis* (Wrapping) | 0.00±0.00z | 3.47±0.43 wxy | 5.52±0.24 o-t | 5.73±0.22 o-s | 6.48±0.19 no | 6.23±0.25 n-q | 6.45±0.39 no | 5.01±0.26 f |
| *S. endus* (Dipping) | 0.00±0.00z | 3.29±0.38 xy | 4.64±0.23 s-v | 6.27±0.35 nop | 7.25±0.3 lmn | 7.24±0.05 lmn | 8.06±0.1 jkl | 5.44±0.21 e |
| *S. endus* (Wrapping) | 0.00±0.00z | 4.31±0.86 u-x | 6.6±0.66 mno | 6.98±0.16 lmn | 8.76±0.56 ijk | 9.11±0.15 ij | 9.57±0.19 hi | 6.66±0.38 d |
| Time points (Mean±SD) | 0.00±0.00 g | 4.61±0.56 f | 7.08±0.3 e | 8.44±0.25 d | 9.69±0.29 c | 10.51±0.21 b | 11.29±0.21 a | |
| $p_{\text{Treatment}}$ | < 0.0001 | | | | | | | |
| $p_{\text{Time}}$ | < 0.0001 | | | | | | | |
| $p_{\text{Treatment × Time}}$ | < 0.0001 | | | | | | | |
| **2nd Trial** | | | | | | | | |
| Control | 0.00±0.00y | 8.18±0.49 mn | 12.4±0.12 ghi | 14.3±0.18 ef | 16.23±0.28 cd | 18.95±0.32 b | 20.7±0.16 a | 13.11±0.24 a |
| Fungicide "Nativo" | 0.00±0.00y | 5.88±0.24 qi | 9.72±0.16 kl | 12.76±0.22 gh | 14.28±0.11 ef | 15.49±0.28 de | 17.06±0.37 c | 10.96±0.21 b |
| $CaCL_2$ | 0.00±0.00y | 5.33±0.83 qrs | 8.85±0.66 lm | 11.25±0.21 ij | 12.38±0.1 ghi | 14.15±0.07 ef | 15.45±0.18 de | 9.85±0.32 c |
| *B. subtilis* (Dipping) | 0.00±0.00y | 2.89±0.37 vw | 3.62±0.47 uv | 4.01±0.23 s-v | 4.78±0.04 r-u | 4.62±0.17 r-u | 4.36±0.37 stu | 3.66±0.26 g |
| *B. subtilis* (Wrapping) | 0.00±0.00y | 2.74±1.41 vwx | 4.38±0.36 stu | 5.04±0.48 rst | 6.53±0.16 opq | 7.95±0.15 mn | 7.85±0.16 mno | 5.18±0.42 f |
| *S. endus* (Dipping) | 0.00±0.00y | 3.75±0.56 t-v | 4.96±0.59 r-u | 6.5±0.2 pq | 8.03±0.27 mn | 9.87±0.15 kl | 10.65±0.22 jk | 6.48±0.29 e |
| *S. endus* (Wrapping) | 0.00±0.00y | 4.72±0.78 r-u | 7.49±0.85 opq | 8.08±0.14 mn | 10.33±0.62 jk | 12.22±0.2 hi | 13.67±0.16 fg | 8.29±0.4 d |
| Time points (Mean±SD) | 0.00±0.00 g | 4.78±0.67 f | 7.35±0.46 e | 8.85±0.24 d | 10.36±0.23 c | 11.89±0.19 b | 12.82±0.23 a | |
| $p_{\text{Treatment}}$ | < 0.0001 | | | | | | | |
| $p_{\text{Time}}$ | < 0.0001 | | | | | | | |
| $p_{\text{Treatment × Time}}$ | < 0.0001 | | | | | | | |

Values denote the mean ± standard deviation (means ± SD) of three biological replicates per treatment.
Different letters signify statistically significant differences among treatments using Tukey's Honestly Significant Difference (HSD) Test based on the *p*-value of treatments ($p_{\text{Treatment}} < 0.05$), time ($p_{\text{Time}} < 0.05$), or their interaction ($p_{\text{Treatment×Time}} < 0.05$).

**Table S3. Effect of the cell-free culture filtrates of bacterial bioagents *B. subtilis* and *S. endus* on the titratable acidity (%) caused by *B. cinerea*, the causal agent of gray mold disease on apple fruits over 90 days storage under cold storage.**

| Treatment | Days post-storage (dps) | | | | | | | Treatments (Mean±SD) |
|---|---|---|---|---|---|---|---|---|
| | 0 | 15 | 30 | 45 | 60 | 75 | 90 | |
| **1st Trial** | | | | | | | | |
| Control | 1.43±0.02 abc | 1.44±0.03ab | 1.31±0.06a-f | 1.26±0.09a-h | 1.20±0.06a-i | 1.13±0.03b-k | 1.13±0.11b-k | 1.27±0.06a |
| Fungicide "Nativo" | 1.46±0.07 a | 1.42±0.16abc | 1.18±0.07a-j | 1.11±0.14c-l | 1.09±0.10d-l | 0.95±0.08h-p | 0.93±0.10i-p | 1.16±0.10b |
| $CaCL_2$ | 1.44±0.04 abc | 1.18±0.19a-i | 1.01±0.07f-n | 1.05±0.04e-m | 0.96±0.11h-o | 0.89±0.08i-q | 0.85±0.07j-q | 1.05±0.08c |
| *B. subtilis* (Dipping) | 1.42±0.02 abc | 1.12±0.11b-k | 0.85±0.08k-q | 0.75±0.09m-q | 0.63±0.08opq | 0.62±0.15pq | 0.58±0.13q | 0.85±0.09d |
| *B. subtilis* (Wrapping) | 1.41±0.08 a-d | 1.15±0.18a-k | 1.05±0.03e-m | 0.97±0.15g-n | 0.94±0.11h-p | 0.75±0.07m-q | 0.63±0.03opq | 0.99±0.09c |
| *S. endus* (Dipping) | 1.39±0.05 a-d | 1.29±0.18a-g | 1.01±0.07f-n | 0.91±0.14i-p | 0.79±0.03l-q | 0.76±0.08m-q | 0.69±0.11n-q | 0.98±0.09c |
| *S. endus* (Wrapping) | 1.36±0.06a-e | 1.26±0.07a-h | 1.05±0.03e-m | 1.00±0.07f-n | 0.84±0.03k-q | 0.71±0.20n-q | 0.75±0.05m-q | 1.00±0.07c |
| Time points (Mean±SD) | 1.42±0.05 a | 1.27±0.13 b | 1.07±0.06 c | 1.01±0.10 cd | 0.92±0.07 de | 0.83±0.10 ef | 0.79±0.08 f | |
| $p_{Treatment}$ | < 0.0001 | | | | | | | |
| $p_{Time}$ | < 0.0001 | | | | | | | |
| $p_{Treatment \times Time}$ | =0.0103 | | | | | | | |
| **2nd Trial** | | | | | | | | |
| Control | 1.47±0.03 a | 1.45±0.08a | 1.33±0.07a-d | 1.28±0.02 a-g | 1.26±0.02 a-h | 1.14±0.02 b-k | 1.04±0.03 d-n | 1.28±0.03 a |
| Fungicide "Nativo" | 1.44±0.04 ab | 1.46±0.19a | 1.28±0.02a-g | 1.20±0.07a-j | 1.12±0.02c-k | 0.90±0.10 j-q | 0.86±0.12 k-s | 1.18±0.08 b |
| $CaCL_2$ | 1.41±0.07 abc | 1.21±0.13a-i | 0.99±0.05f-o | 1.07±0.13d-l | 0.96±0.07 h-p | 0.87±0.07 k-r | 0.56±0.03 rs | 1.01±0.08 c |
| *B. subtilis* (Dipping) | 1.44±0.09 ab | 0.98±0.11g-o | 0.80±0.05 l-s | 0.70±0.08 o-s | 0.66±0.08 p-s | 0.56±0.08 s | 0.56±0.05 rs | 0.81±0.08 d |
| *B. subtilis* (Wrapping) | 1.40±0.07 abc | 1.13±0.18c-k | 1.05±0.05d-m | 0.99±0.16 f-o | 0.91±0.05 i-q | 0.76±0.08 m-s | 0.66±0.11 qrs | 0.98±0.10 c |
| *S. endus* (Dipping) | 1.47±0.13 a | 1.31±0.09a-e | 0.99±0.20 f-o | 0.92±0.11 i-q | 0.78±0.05 l-s | 0.73±0.07 n-s | 0.71±0.13 o-s | 0.99±0.11 c |
| *S. endus* (Wrapping) | 1.42±0.07 abc | 1.30±0.04a-f | 1.03±0.08 d-n | 1.00±0.07 e-o | 0.85±0.08 k-s | 0.71±0.15 o-s | 0.76±0.08 m-s | 1.01±0.08 c |
| Time points (Mean±SD) | 1.43±0.07 a | 1.26±0.12 b | 1.07±0.07 c | 1.02±0.09 c | 0.93±0.05 d | 0.81±0.08 e | 0.74±0.08 e | |
| $p_{Treatment}$ | < 0.0001 | | | | | | | |
| $p_{Time}$ | < 0.0001 | | | | | | | |
| $p_{Treatment \times Time}$ | < 0.0001 | | | | | | | |

Values denote the mean ± standard deviation (means ± SD) of three biological replicates per treatment.
Different letters signify statistically significant differences among treatments using Tukey's Honestly Significant Difference (HSD) Test based on the *p*-value of treatments ($p_{Treatment} < 0.05$), time ($p_{Time} < 0.05$), or their interaction ($p_{Treatment \times Time} < 0.05$).

**Table S4. Effect of the cell-free culture filtrates of bacterial bioagents *B. subtilis* and *S. endus* on the firmness (Newton) caused by *B. cinerea*, the causal agent of gray mold disease on apple fruits over 90 days storage under cold storage.**

| Treatment | Days post-storage (dps) | | | | | | | Treatments |
|---|---|---|---|---|---|---|---|---|
| | 0 | 15 | 30 | 45 | 60 | 75 | 90 | (Mean±SD) |
| **1st Trial** | | | | | | | | |
| Control | 14.54±0.34 ab | 09.85±1.78 f-l | 08.14±0.67 k-o | 07.37±0.28 l-o | 06.41±0.25 mno | 06.39±0.14 no | 05.50±0.41 o | 08.31±0.55 c |
| Fungicide "Nativo" | 14.40±0.14 abc | 14.49±2.22 ab | 12.89±0.57a-g | 11.82±0.37 a-i | 11.39±1.17 b-k | 10.96±0.69 d-k | 10.14±1.38 f-l | 12.30±0.93 a |
| $CaCL_2$ | 14.73±0.68 a | 12.82±1.60 a-g | 11.83±0.58 a-i | 11.17±1.04 c-k | 11.17±0.58 c-k | 08.67±0.76 i-o | 08.50±0.50 j-o | 11.27±0.82 b |
| *B. subtilis* (Dipping) | 14.33±0.25 abc | 14.33±0.29 abc | 14.33±0.76 abc | 13.00±0.50 a-f | 12.33±0.76 a-h | 11.83±1.26 a-i | 11.67±1.04 a-j | 13.12±0.70 a |
| *B. subtilis* (Wrapping) | 14.53±0.25 ab | 13.65±2.00 a-e | 10.67±1.44 e-k | 09.00±0.50 i-n | 09.67±1.26 g-m | 09.50±1.32 h-n | 09.33±0.76 h-n | 10.91±1.08 b |
| *S. endus* (Dipping) | 14.30±0.26 abc | 14.17±1.89 a-d | 09.67±1.15 g-m | 09.17±0.58 h-n | 09.83±0.76 f-l | 09.67±0.29 g-m | 09.00±1.80 i-n | 10.83±0.96 b |
| *S. endus* (Wrapping) | 14.39±0.20 abc | 11.33±0.76 b-k | 11.00±0.50 d-k | 09.83±0.55 f-l | 10.00±0.87f-l | 09.33±1.04 h-n | 09.03±0.87 i-n | 10.70±0.69 b |
| Time points (Mean±SD) | 14.46±0.30 a | 12.95±1.51 b | 11.22±0.81 c | 10.19±0.55 d | 10.11±0.81 d | 9.48±0.79 de | 9.02±0.97 e | |
| $p_{Treatment}$ | < 0.0001 | | | | | | | |
| $p_{Time}$ | < 0.0001 | | | | | | | |
| $p_{Treatment \times Time}$ | < 0.0001 | | | | | | | |
| **2nd Trial** | | | | | | | | |
| Control | 14.83±0.29 a | 09.67±0.58 h-n | 08.50±0.50 k-p | 07.83±0.29 m-p | 07.40±0.53 nop | 06.83±0.29 op | 06.17±0.29 p | 08.75±0.39 a |
| Fungicide "Nativo" | 15.07±0.60 a | 13.67±2.31 a-d | 12.67±0.58 a-f | 12.00±0.50 b-h | 11.00±0.50 d-k | 09.83±0.29 g-n | 09.83±1.04 g-n | 12.01±0.83 b |
| $CaCL_2$ | 14.97±0.45 a | 13.83±1.61 abc | 11.50±1.32 c-i | 11.50±0.87 c-i | 10.50±0.87 e-m | 08.67±0.29 j-p | 08.07±0.12 l-p | 11.29±0.79 c |
| *B. subtilis* (Dipping) | 15.00±0.50 a | 14.50±0.50 ab | 13.83±1.26 abc | 13.17±0.29 a-e | 12.50±0.50 a-g | 12.00±0.00 b-h | 11.37±0.32 c-j | 13.20±0.48 de |
| *B. subtilis* (Wrapping) | 14.93±0.40 a | 13.67±0.58 a-d | 11.17±0.76 c-k | 09.83±0.29 g-n | 09.40±0.53 h-o | 08.70±0.26 j-p | 08.17±0.76 l-p | 10.84±0.51 cd |
| *S. endus* (Dipping) | 15.13±0.71 a | 13.17±2.36 a-e | 10.33±1.53 f-n | 09.83±0.76 g-n | 09.33±1.15 h-o | 09.23±0.25 i-o | 08.50±0.50 k-p | 10.79±1.04 ef |
| *S. endus* (Wrapping) | 14.90±0.36 a | 11.33±0.58 c-j | 10.67±0.58 e-l | 10.03±0.05 f-n | 09.20±0.72 i-o | 09.00±0.50 i-o | 08.53±0.52 k-p | 10.52±0.46 f |
| Time points (Mean±SD) | 14.98±0.47 a | 12.83±1.22 b | 11.24±0.93 c | 10.60±0.43 cd | 09.90±0.69 de | 09.18±0.27 ef | 8.66±0.51 f | |
| $p_{Treatment}$ | < 0.0001 | | | | | | | |
| $p_{Time}$ | < 0.0001 | | | | | | | |
| $p_{Treatment \times Time}$ | < 0.0001 | | | | | | | |

Values denote the mean ± standard deviation (means ± SD) of three biological replicates per treatment.
Different letters signify statistically significant differences among treatments using Tukey's Honestly Significant Difference (HSD) Test based on the *p*-value of treatments ($p_{Treatment} < 0.05$), time ($p_{Time} < 0.05$), or their interaction ($p_{Treatment \times Time} < 0.05$).

**Table S5. Effect of the cell-free culture filtrates of bacterial bioagents *B. subtilis* and *S. endus* on the total soluble solids (°Brix) caused by *B. cinerea*, the causal agent of gray mold disease on apple fruits over 90 days storage under cold storage.**

| Treatment | Days post-storage (dps) | | | | | | | Treatments (Mean±SD) |
|---|---|---|---|---|---|---|---|---|
| | 0 | 15 | 30 | 45 | 60 | 75 | 90 | |
| **1st Trial** | | | | | | | | |
| Control | 8.43±0.15 m-p | 12.33±0.42 c-f | 13.53±0.42 a-d | 13.60±0.53 a-d | 13.67±0.58 abc | 14.07±0.51ab | 14.80±0.20 a | 12.92±0.40 a |
| Fungicide “Nativo” | 8.43±0.40 m-p | 10.92±0.38 e-j | 10.99±0.64 e-i | 11.40±0.58 e-h | 12.34±1.27c-f | 12.59±1.33 b-e | 13.18±0.32 a-d | 11.40±0.70 b |
| $CaCL_2$ | 8.27±0.31 op | 10.60±0.53 g-k | 10.67±0.58 f-j | 10.80±0.35 f-j | 10.87±0.42 f-j | 11.47±0.50 e-h | 11.93±0.12 d-g | 10.66±0.40 c |
| *B. subtilis* (Dipping) | 8.38±0.11 m-p | 08.23±0.23 op | 08.14±0.92 p | 08.51±0.75 m-p | 08.96±0.38 k-p | 09.38±0.90 i-p | 10.03±0.56 h-m | 08.80±0.55 e |
| *B. subtilis* (Wrapping) | 8.37±0.32 m-p | 08.80±0.20 l-p | 08.93±0.12 k-p | 09.87±0.12 h-o | 10.02±0.05 h-n | 10.47±0.53 g-l | 10.60±0.53 g-k | 09.58±0.25 d |
| *S. endus* (Dipping) | 8.33±0.12 nop | 09.27±0.31 j-p | 09.33±0.42 i-p | 09.40±0.53 i-p | 09.80±0.40 h-p | 10.27±0.46 g-l | 10.33±0.31 g-l | 09.53±0.36 d |
| *S. endus* (Wrapping) | 8.40±0.20 m-p | 09.53±0.23 i-p | 09.80±0.35 h-p | 10.00±0.20 h-n | 10.20±0.20h-l | 10.47±0.42 g-l | 10.60±0.53 g-k | 09.86±0.30 d |
| Time points (Mean±SD) | 8.37±0.23 f | 09.95±0.33 e | 10.20±0.49 de | 10.51±0.43 cd | 10.83±0.46 bc | 11.24±0.66 ab | 11.64±0.37 a | |
| $p_{Treatment}$ | < 0.0001 | | | | | | | |
| $p_{Time}$ | < 0.0001 | | | | | | | |
| $p_{Treatment \times Time}$ | < 0.0001 | | | | | | | |
| **2nd Trial** | | | | | | | | |
| Control | 8.27±0.21 yz | 11.73±0.64 g-k | 13.53±0.23 bcd | 13.80±0.35 bc | 13.87±0.12 bc | 14.13±0.31 ab | 14.87±0.12 a | 12.89±0.28 a |
| Fungicide “Nativo” | 8.30±0.26 yz | 10.67±0.40 m-q | 11.70±0.35 g-l | 12.23±0.12 f-i | 12.63±0.42 d-g | 12.97±0.31 c-f | 13.30±0.20 b-e | 11.69±0.29 b |
| $CaCL_2$ | 8.27±0.21 yz | 10.63±0.23 m-q | 11.23±0.12 j-o | 11.43±0.12 i-n | 11.50±0.20 h-m | 12.03±0.23 f-j | 12.43±0.12e-h | 11.08±0.17 c |
| *B. subtilis* (Dipping) | 8.17±0.06 yz | 08.20±0.53 yz | 08.53±0.42 xyz | 08.80±0.35 v-z | 08.87±0.12 v-z | 09.13±0.12 u-y | 09.60±0.35 r-w | 08.76±0.28 f |
| *B. subtilis* (Wrapping) | 8.07±0.15 z | 08.67±0.31 w-z | 08.87±0.12 v-z | 09.73±0.31 q-v | 10.07±0.12 p-u | 10.47±0.42 n-s | 10.80±0.20 k-p | 09.52±0.23 e |
| *S. endus* (Dipping) | 8.43±0.40 xyz | 09.00±0.40 v-z | 09.40±0.35 t-x | 09.73±0.31 q-v | 10.13±0.12 p-t | 10.27±0.33 o-t | 10.67±0.12 m-q | 09.66±0.28 de |
| *S. endus* (Wrapping) | 8.23±0.06 yz | 09.40±0.35 t-x | 09.53±0.31 s-w | 10.00±0.20 p-u | 10.27±0.23 o-t | 10.53±0.35 m-r | 10.73±0.46 l-p | 09.81±0.27 d |
| Time points (Mean±SD) | 8.25±0.19 f | 09.76±0.41 e | 10.40±0.27 d | 10.82±0.25 c | 11.05±0.19 c | 11.36±0.28 b | 11.77±0.22 a | |
| $p_{Treatment}$ | < 0.0001 | | | | | | | |
| $p_{Time}$ | < 0.0001 | | | | | | | |
| $p_{Treatment \times Time}$ | < 0.0001 | | | | | | | |

Values denote the mean ± standard deviation (means ± SD) of three biological replicates per treatment.
Different letters signify statistically significant differences among treatments using Tukey's Honestly Significant Difference (HSD) Test based on the *p*-value of treatments ($p_{Treatment} < 0.05$), time ($p_{Time} < 0.05$), or their interaction ($p_{Treatment \times Time} < 0.05$).

**Table S6. Sequences from different bacterial genera that produce significant alignment with 16S ribosomal RNA gene from *Bacillus subtilis* [a,b]**

| Description | Max Score | Total Score | Query Cover (%) | E value | Identity ( %) | Accession Length (bp) | Accession |
|---|---|---|---|---|---|---|---|
| *Bacillus subtilis* strain Inu 16S ribosomal RNA gene, partial sequence | 1166 | 1166 | 100 | 0.0 | 94.14 | 1315 | OK444102.1 |
| *Bacillus subtilis* subsp. inaquosorum strain SS2-10 16S ribosomal RNA gene, partial sequence | 1155 | 1155 | 90 | 0.0 | 96.70 | 1397 | MK373052.1 |
| *Bacillus subtilis* strain T3 16S ribosomal RNA gene, partial sequence | 1155 | 1155 | 88 | 0.0 | 97.50 | 1001 | MH512934.1 |
| *Bacillus subtilis* strain GB16 16S ribosomal RNA gene, partial sequence | 1155 | 1155 | 90 | 0.0 | 96.55 | 1512 | AY911607.1 |
| *Bacillus subtilis* strain MEB63 16S ribosomal RNA gene, partial sequence | 1153 | 1153 | 90 | 0.0 | 96.56 | 786 | MT415745.1 |
| *Bacillus subtilis* strain A-SRETCR 16S ribosomal RNA gene, partial sequence | 1151 | 1151 | 90 | 0.0 | 96.55 | 1143 | KU736839.1 |
| *Bacillus flexus* strain psrm 16S ribosomal RNA gene, partial sequence | 1151 | 1151 | 90 | 0.0 | 96.56 | 810 | KC964543.1 |
| *Bacillus tequilensis* strain SM-43 16S ribosomal RNA gene, partial sequence | 1149 | 1149 | 90 | 0.0 | 96.55 | 1407 | MT377888.1 |
| *Bacillus subtilis* strain CC5 16S ribosomal RNA gene, partial sequence | 1149 | 1149 | 90 | 0.0 | 96.55 | 1015 | MK720674.1 |
| *Bacillus* sp. (in: Bacteria) strain CP_E1 16S ribosomal RNA gene, partial sequence | 1149 | 1149 | 90 | 0.0 | 96.55 | 1001 | MN880211.1 |
| *Bacillus subtilis* strain DS1620 16S ribosomal RNA gene, partial sequence | 1149 | 1149 | 90 | 0.0 | 96.55 | 1392 | MN818641.1 |
| *Bacillus subtilis* strain SA 82 16S ribosomal RNA gene, partial sequence | 1149 | 1149 | 90 | 0.0 | 96.55 | 1403 | KY194723.1 |
| *Bacillus* sp. (in: Bacteria) strain ZHH-2 16S ribosomal RNA gene, partial sequence | 1149 | 1149 | 90 | 0.0 | 96.55 | 1413 | MH290497.1 |
| *Bacillus* sp. (in: Bacteria) strain XZ-139 16S ribosomal RNA gene, partial sequence | 1149 | 1149 | 90 | 0.0 | 96.55 | 788 | MF059112.1 |
| *Bacillus subtilis* strain BR19 16S ribosomal RNA gene, partial sequence | 1149 | 1149 | 90 | 0.0 | 96.55 | 1468 | KX179630.1 |
| *Bacillus subtilis* strain Db1 16S ribosomal RNA gene, partial sequence | 1149 | 1149 | 90 | 0.0 | 96.55 | 843 | KU198922.1 |
| *Bacillus subtilis* strain MA-58 16S ribosomal RNA gene, partial sequence | 1149 | 1149 | 90 | 0.0 | 96.55 | 1038 | KX426658.1 |
| *Bacillus subtilis* strain Bs_Asi 16S ribosomal RNA gene, complete sequence | 1149 | 1149 | 90 | 0.0 | 96.55 | 1417 | KP864637.1 |
| *Bacillus* sp. XY7 16S ribosomal RNA gene, partial sequence | 1149 | 1149 | 90 | 0.0 | 96.55 | 1410 | KF986309.1 |

[a] The listed bacterial genera were identified using the nucleotide-nucleotide BLAST (BLASTn) (Altschul et al. 1997, 2005) using *B. subtilis* —Isolate AYA2023" (GenBank Accession No. OR271987 ; 767 bp) as a query sequence against available data in GenBank, national center for biotechnology information website (NCBI, http://www.ncbi.nlm.nih.gov/gene/).

[b] Listed bacterial genera were used to generate the phylogenetic tree presented in Figure 1.
